# Modeling Fixed Bed Membrane Reactors for Hydrogen Production through Steam Reforming Reactions: A Critical Analysis

**DOI:** 10.3390/membranes8020034

**Published:** 2018-06-19

**Authors:** Maria Anna Murmura, Stefano Cerbelli, Maria Cristina Annesini

**Affiliations:** Dipartimento di Ingegneria Chimica Materiali Ambiente, Sapienza Università Di Roma, Via Eudossiana 18, 00184 Roma, Italy; stefano.cerbelli@uniroma1.it (S.C.); annesini@uniroma1.it (M.C.A.)

**Keywords:** membrane reactor, hydrogen, Pd-based membranes, CFD, reactor modeling

## Abstract

Membrane reactors for hydrogen production have been extensively studied in the past years due to the interest in developing systems that are adequate for the decentralized production of high-purity hydrogen. Research in this field has been both experimental and theoretical. The aim of this work is two-fold. On the one hand, modeling work on membrane reactors that has been carried out in the past is presented and discussed, along with the constitutive equations used to describe the different phenomena characterizing the behavior of the system. On the other hand, an attempt is made to shed some light on the meaning and usefulness of models developed with different degrees of complexity. The motivation has been that, given the different ways and degrees in which transport models can be simplified, the process is not always straightforward and, in some cases, leads to conceptual inconsistencies that are not easily identifiable or identified.

## 1. Introduction

Membrane reactors (MRs) have received significant attention for their potential use in decentralized hydrogen production systems, allowed by the integrated production and separation of hydrogen [1,2,3]. The reactions most commonly carried out are those of steam reforming of different carbon-based feeds such as methane [4,5,6,7,8,9,10,11,12,13,14], methanol [15,16,17,18,19,20,21], ethanol [22,23,24,25,26,27], biogas [28,29], and glycerol [30,31,32]; water-gas shift [33,34,35,36,37,38]; ammonia decomposition [39,40]; and the dehydrogenation of alkanes [41,42,43,44]. In all cases the equilibrium of the reaction is shifted by removing hydrogen through a membrane. Although the use of porous ceramic membranes has also been proposed [16,17,45], dense Pd-based membranes are currently those most commonly envisaged for the selective removal of hydrogen, and therefore only the latter will be considered in what follows. The present work is focused on steam reforming reactions, but most observations may be extended to other reacting systems. Research on MRs has been both experimental and theoretical, with the development of models that could accurately capture the different phenomena taking place in the reactors and affecting their performance. The complexity of membrane reactors may be recognized even by merely considering the number of parameters involved in their design, including reactor configuration and dimensions, inlet flow rate and composition, pressure, temperature, membrane permeability, catalyst activity, heating system, and sweep gas composition and direction of flow.

The aim of this work is not only to present the work that has been carried out in the past years on membrane reactor modeling, but also to shed some light on the meaning and significance of developing models with different degrees of complexity. To do so, a few preliminary considerations are necessary. The choice of the degree of complexity may be motivated by different reasons, including the main objective for which the model is being developed, the system that needs to be described, and the computational cost that one is willing or capable to endure. Before we look into these three facets, we briefly consider the phenomena taking place in membrane reactors. Broadly speaking, they may be identified as

1. Momentum transport
2. Mass transport by convection, dispersion and permeation across the membrane of different components which are produced/consumed by chemical reactions
3. Energy transport by convection, conduction, exchange between permeate and retentate, and exchange between the reactor and its wall, as well as the heat generation due to the chemical reactions

Note that in principle, all these phenomena are interrelated.

Several choices may be made when tackling steam reforming in membrane reactors, the most conceptually simple of which is the construction of an extremely complete model, accounting for all three transport phenomena and without the introduction of any simplifying assumption. The advantage of this approach is that, provided that the model is correctly written, the results must necessarily be right if the parameters are chosen correctly. The disadvantages are numerous. To begin with, the model may turn out to be useless, in that the elements of complexity are so numerous that making sense of the relevant physics becomes close to impossible and predictions can only be made by solving it in its entirety any time one of the conditions changes. In addition, although the correct writing of the model equations in the absence of simplifying assumptions truly is simple, unavoidable approximations are associated with (i) the presence of a disordered media that provides the support for the catalyst, and (ii) the use of empirical correlations for the evaluation of several of the model parameters required. Having said this, two approaches are generally possible to determine the desired degree of complexity and of coupling between the different equations of change:

1. a top-down approach, where the complete model is gradually simplified by removing the description of all phenomena that can be considered to be unimportant
2. a bottom-up approach, in which the most simple model is initially considered, and all the significant phenomena are gradually added

Next, we try to better define the idea of “simple” models. The complexity of a model may be determined on the basis of:

1. the transport phenomena described (e.g., isothermal vs. non-isothermal models)
2. the dimension of the problem (e.g., 1D vs. 2D models)
3. the transport mechanisms considered (e.g., neglecting dispersion with respect to convection)
4. the detail used in the description of each mechanism (e.g., assuming the same diffusion coefficient for all components vs. accounting for differences in diffusion coefficient values)

In addition, “mixed” degrees of complexity may be adopted. For instance, one may write a 1D momentum balance equation with a 2D energy balance equation; or account for mass dispersion in the radial direction and mass convection in the axial direction.

Generally, the complexity should be chosen so as to obtain the desired result with the lowest possible cost, in terms of both computational cost and of information required. This leads us back to our original observation that there may exist several different objectives. If, for example, one wanted to gain insight into the influence of different mass transport mechanisms on the performance of the system, it may be reasonable to develop several simple models that allow the easy comprehension of the response of the system to changes in a single transport mechanism, and later a more complex model providing information on the interplay between the different mechanism and whose interpretation is favored by the previous understanding of the more simple models. On the other hand, if the aim were to describe an existing apparatus, it may be reasonable to determine which transport phenomena and mechanisms are significant under the conditions of interest, for example through the definition of dimensionless parameters, and develop a model containing only the necessary elements.

Given the different ways, degrees, and reasons for the simplification of transport models, their classification is particularly challenging. Here, we review modeling of membrane reactors according to the following approach: we begin by describing commonly used reactor configurations (Section 2) and defining the parameters commonly used to describe the performance of membrane reactors (Section 3). In Section 4, Section 5 and Section 6 we discuss the equations of change and how they differ in the 1D and 2D cases. In Section 7 we present the constitutive equations needed to describe the rates of reaction, hydrogen permeation across the membrane, and heat exchange with the reactor wall and permeate. In Section 8 and Section 9 some 1D and 2D models presented in the literature are discussed. Finally, Section 10 presents models with different degrees of complexity, in which authors have explicitly dealt with the problem of how to choose the most appropriate forms of the transport models. This last section also provides an opportunity to discuss the coupling of transport equations.

## 2. Reactor Configurations

**Figure 1:**
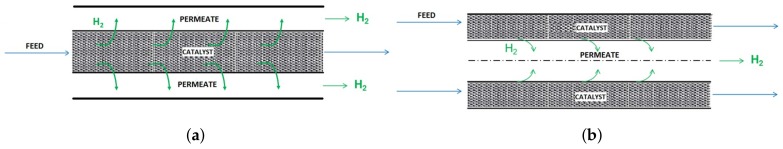
Shell and tube membrane reactor configuration with (**a**) catalyst in the inner tube and sweep gas flowing in the outer annular volume and (**b**) catalyst in the outer annular volume and sweep gas flowing in the inner tube. Co-current configurations have been shown as examples.

Membrane reactors may be divided into two main categories, packed bed reactors and fluidized bed reactors. In the first case, they generally present a shell and tube configuration. The catalyst may be placed either in the inner cylindrical volume (Figure 1a) or, more commonly, in the outer annulus (Figure 1b), and the permeate flows in the remaining section. In both cases, the additional choice to be made is the direction of flow of the sweep gas with respect to the reacting gas mixture. The counter-current configuration is most commonly used, although particular attention has to be placed in avoiding the back permeation of hydrogen in the region close to the feed inlet, where the hydrogen concentration of the retentate may be lower than that in the permeate. Naturally this problem is reduced if the two volumes are kept at different pressures. A more thorough discussion on the characterization of the permeate flow rate is reported in Section 7.2.

These configurations are those most commonly used in lab-scale applications. Larger reactors generally make use of multiple tubes, where once again two solutions are possible: either the catalyst is placed in the shell, with the permeate flowing inside the tubes, or the catalyst is placed in the inner volume of the tubes and the permeate volume is represented by the shell.

Packed bed reactors may also be realized as micro-reactors, which have been proposed to reduce heat and mass transfer resistances. The design, simulating that of a plate heat exchanger, consists of an alternation of channels in which the reaction takes place with channels into which hydrogen permeates [46,47,48].

In all the cases discussed, the catalyst may be supported either on pellets or on solid foams. The use of the latter has been proposed in the past years because they present they following advantages [49,50,51,52]

- lower pressure drops due to a void fraction that is almost twice the value achieved in traditional packed beds;
- high surface area to volume ratios, leading to enhanced rates of heat and mass transfer and therefore higher reactions effectiveness factors;
- increased turbulence and convective heat transfer

These advantages are particularly significant in membrane reactors. Lower pressure drops allow to sustain a high driving force for hydrogen permeation, which is essential for the system’s efficiency. The increased heat transfer is important both to favor the kinetics and because reforming reactions are highly endothermic, and although the equilibrium of the reaction is shifted towards the products by means of the hydrogen permeation, reducing heat losses between the heating system and the reaction volume contributes to maintaining a higher equilibrium conversion.

The second configuration is that of a fluidized bed membrane reactor, in which the hydrogen-permeable membrane is immersed in the catalytic bed, operated in either the bubbling or turbulent regime. The main advantage of this type of reactor is represented by the possibility of using smaller catalyst particles, thereby reducing the mass and heat transfer limitations. In fact, fluidized membrane reactors can achieve the same hydrogen flux with a smaller membrane area compared to packed bed membrane reactors [53]. In addition, fluidized bed reactors are capable of operating under isothermal conditions even in the presence of reactions accompanied by strong thermal effects. This makes their use particularly interesting for the autothermal reforming process. On the other hand, fluidized bed reactors tend to require a larger reaction volume to achieve the same production of hydrogen and the membranes must be specifically designed to avoid mechanical deterioration due to the catalyst fluidization.

The remaining part of this work will be focused on fixed-bed membrane reactors.

## 3. Description of Reactor Performance

The performance of membrane reactors may be described in terms of several parameters, all of which are interrelated.

- Reactant conversion: as in the case of traditional reactors, this parameter measures the extent of completion of the reaction. In the case of membrane reactors, their efficiency may be quantified by determining the excess conversion with respect to the equilibrium value that one would obtain from the feed at the same temperature and pressure conditions, as was done, for example, in [23].
- Permeate flow rate: this is a measure of the amount of pure hydrogen produced and is often the design parameter. The value of the total permeate flow rate intrinisically accounts for the efficiency of both the separation and the reaction, albeit without providing information on the relative importance of the two.
- Yield: the yield is generally defined as the ratio between the amount of pure hydrogen produced, i.e., the permeate, and the reactant feed flow rate. Its significance is esentially the same as that of the permeate flow rate with the additional advantage that it allows an easier comparison of systems characterized by different feed flow rates. The maximum value that it may reach is the stoichiometric ratio between hydrogen and the reference reactant.
- Recovery: the recovery is defined as the ratio between the amount of pure hydrogen permeating across the membrane and the amount of hydrogen produced by the reaction. This parameter is, in effect, a measure of the efficiency of the membrane separation in that it provides no information on the extent of reaction completion. Its upperbound is 1 if no hydrogen is present in the feed.
- Separator-based yield: This parameter has been introduced in [54,55] and is defined as the ratio between the hydrogen permeate flow rate and the inlet hydrogen flow rate. Naturally, this parameter can only be defined if hydrogen is already present in the feed and is particularly significant if the feed is the product of a pre-reactor in which equilibrium conditions have been reached. Under these conditions, its maximum value is the inverse of the equilibrium conversion of the reactant. This parameter is particularly interesting because, similarly to the permeate flow rate or yield, it provides information on the efficiency of both reaction and separation, while giving some insight into the relative weight of the two. In the absence of a reaction its maximum value would be equal to 1, and the degree with which it exceeds the value of 1 is indicative of the extent of the reaction.

## 4. Equations of Change

The aim of this section is to present an overview of the mass, momentum, and heat balance equations in the retentate. In all three cases we start from the complete formulation of the problem. The corresponding boundary conditions are also reported (see Table 1, Table 2, Table 3 and Table 4). The momentum, mass, and energy transport equations are discussed individually in their rigorous formulation, without taking into account the coupling between the different phenomena. The simplifications of these equations will be considered later.

As mentioned earlier, we consider two possible reactor geometries, both consisting of a shell and tube configuration but differing in the position of the catalyst. In the most commonly adopted system, the permeate flows in the innermost cylindrical volume and the catalyst is placed in the outer annular volume. The selective Pd layer of membrane is supported on the outer wall of the innermost tube. In fewer applications the catalyst is placed in the cylindrical volume and the permeate flows in the outer shell, with the Pd layer supported on the inner wall of the innermost tube. The location of the membrane is chosen on the basis of mechanical considerations: it is advisable for the membrane to be located on the side at higher pressure, i.e., the retentate side, in order to reduce the risk of delamination of the thin selective layer.

In what follows we will use *L* to identify the length of the reactor, $R_{1}$ for the radius of the membrane, and $R_{2}$ for the radius of the impermeable wall. Therefore, in the first configuration, where the catalyst is placed in the annular volume, we will have $R_{1}<R_{2}$, whereas in the second configuration $R_{2}=0$.

Some remarks may be useful in order to recognize both the complexity of the problem, the coupling between different phenomena, and the simplifications that can be introduced.

**Figure 2:**
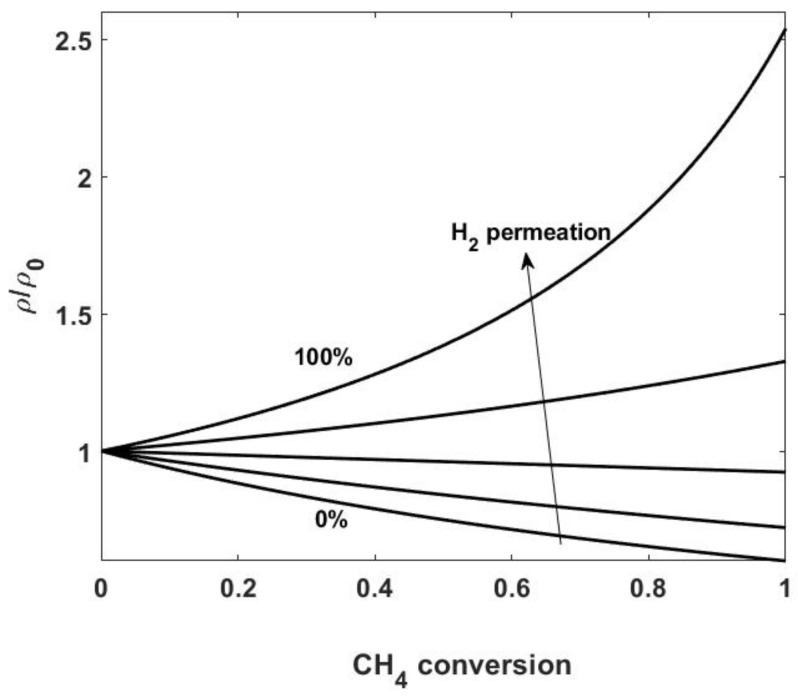
Mass density change as a function of methane conversion in the total reforming reaction at constant temperature and pressure. Top curve refers to total hydrogen permeation, bottom one refers to total reforming without membrane. The other curves refer to 75%, 50% and 25% of the hydrogen removed by the membrane.

- The momentum balance may be described through the modified Navier–Stokes equation for a fixed bed porous medium. The terms on the r.h.s. of Equation (1) refer to (a) pressure gradients, (b) bed permeability and inertial losses, (c) viscous and inertial drag forces imposed by catalyst pore walls on the fluid, and (d) volumetric forces acting on the fluid. The friction coefficient, β, is given by Equation (2) and the stress term is given by Newton’s Equation (3) for a compressible fluid. At the steady state, the momentum balance may simplify to Darcy’s law with the bed permeability expressed through the Kozeny–Carman equation. Works on membrane reactor modeling have adopted both approaches, with a majority resorting to the former (see [1,56,57] and other works by the same authors) rather than the latter (see [7,58,59] and other works by the same authors).
- Mass balance equations are reported in terms of mass units, in order to simplify the coupling with momentum equation. Sometimes, these equations are written assuming the product ρD to be constant. Indeed, the values of the terms $D_{e,r}$ and $D_{e,z}$, appearing in the dispersion tensor, depend on the molecular diffusion coefficient, the gas velocity and the characteristics of the packed bed, while ρ varies as discussed in detail below. A thorough review of transversal and longitudinal dispersion in packed beds is presented in the work of Delgado [60]. As for the boundary conditions, at the reactor inlet two choices are possible: the Danckwerts condition, which meets the requirement of flux continuity, or the condition of concentration continuity. The consensus is larger for the outlet boundary condition, where a purely convective flux is assumed. The radial boundary conditions are of impermeability on one of the walls and hydrogen permeation on the other. For all other components, impermeability is imposed on both walls, assuming that the membrane presents infinite selectivity towards hydrogen. The different forms in which the hydrogen permeating flux may be described are discussed in greater detail in Section 7.2.
- Some authors have proposed the use of a Stefan–Maxwell-like expression to describe the dispersive flux [21], thereby making the implicit assumption that the balance of forces on gas molecules, from which this expression derives, applies to dispersion as well as molecular diffusion. However, it is worth observing that existing theoretical approaches quantifying dispersion in periodic and disordered media are grounded on a diluted assumption for the transported species [61,62,63]. Since semi-empirical correlations used to interpret experimental data are implicitly or explicitly based on these theoretical results, the validity of the Stefan–Maxwell constitutive equation at the Darcy scale should be further investigated.
- In many cases, mass balance equations are written in molar units, as reported in Table 3. It is worth noting that in this case, the mass balance equation may be simplified by assuming cD to be constant, provided that isothermal conditions can be assumed; furthermore from a rigorous point of view, v∗ should be the molar average velocity of the gas mixture, which is, in general, different from the mass average velocity used in the momentum equation. Even if the difference between the velocity may be significant for mixtures containing components with large differences in the molecular weight (in our systems carbon dioxide (MW 44) and hydrogen (MW 2)), it is likely that the errors are of the same order of magnitude of the uncertainties in the evaluation of the dispersion coefficients.
- The reaction rates inserted in the mass balance equation must be intended as effective reaction rates per unit volume of the catalyst bed. Therefore, in the presence of significant intraparticle or interparticle mass or heat transport limitations, a reduction of the efficiency factor must be accounted for. The efficiency factor is explicitly accounted for in some models (see for example [64]). It is worth noting that the characteristic dimension of the catalyst strongly affects the transport limitations, as well as affecting the pressure drop in the catalytic bed, which in turn results in a reduction of the driving force for hydrogen permeation. More specifically, large catalyst sizes result in a low efficiency factor, but also in low head losses in the packed bed.

- To evaluate the possibility of simplifying the mass balance equations, it is useful to discuss how the mass or molar density varies along the reactor, depending on changes in pressure, temperature and gas composition. Usually, in such a reactor, no large head losses occur and pressure is almost constant. Therefore, the molar density, *c*, only depends on temperature changes; in other words, in isothermal or almost isothermal conditions, *c* can be assumed to be constant. As for the mass density, ρ, changes in gas composition result in its variation, even under constant pressure and temperature conditions. These variations can be significant, especially in the reforming process, where low molecular weight compounds (hydrogen) are obtained. We also remark that the situation is completely different for conventional and membrane reactors: in fact, if the hydrogen produced by the reforming remains in the reactor, mass density decreases as the reaction proceeds; on the other hand, in a membrane reactor, where hydrogen is continuously removed as the current flows downstream the reactor, a significant increase in the mass density may be observed (see Figure 2 for methane reforming). Note that in the evaluation of integral quantities, changes in the mass density with composition generally have a negligible effect. On the other hand, by neglecting density changes, the resulting concentration profiles may suffer from inaccuracies that do not enable a correct quantification of effects such as concentration polarization and membrane inhibition (see Section 7.2). The choice of accounting for density changes therefore depends on the scope of the study and should be made on a case-by-case basis.
- As regards the energy transport equation, reported in Table 4, we only remark that terms related to the change of pressure, to the viscous effects, and to the dispersive fluxes are neglected. Values of the effective heat conductivity and of the heat transfer coefficients (appearing in ${\dot{Q}}_{wall}$ and ${\dot{Q}}_{permeate}$) depend on the properties of the gas and of the catalyst bed, and change sizeably depending on whether the catalyst is supported on pellets or solid foams. Several correlations have been reported in the literature [49,65,66] and are discussed more thoroughly in Section 7.3.

The equations that describe transport phenomena in the permeate side are analogous to those presented here, while considering a single phase (without the catalyst) and neglecting all terms attributable to the reactions. Generally, the degree of complexity of the equations describing the permeate is equal to, or more commonly lower than, the one adopted to describe the retentate. In fact, while the retentate is often described through 2D models, the permeate is usually described through 1D—if not simpler—models, although some groups have shown the importance of using 2D models when describing mass transfer in the permeate side of a membrane separator if the trans-membrane flux is high in order to correctly predict hydrogen recovery [67].

The “general” or “complete” model, built by considering all three transport phenomena, is almost intractable without high computational effort and the use of specialized software for finite element analysis; therefore, it is interesting to discuss how less complex models can be derived. Before describing the different models reported in the literature, in the following paragraphs each balance equation is analyzed and different simplified forms used in the literature models are derived, with a discussion of the underlying simplifying assumptions.

## 5. 1D Models

In some cases, 1D models have been used either as a preliminary step towards the development of more complete 2D models, or as simplified descriptions, to be used when the operating conditions fall within a pre-determined range [24,64,68,69]. More commonly, they have been employed to describe experimental results [5,6,23]. The fundamental idea of 1D models is that there is a predominant unidirectional (axial) flow and a description only of the gradients in the axial direction is sufficient for many engineering analyses. To this aim, simple relations can be obtained by integrating the equations of change over a control volume included between the cross-sections at *z* and z+Δz and using the divergence theorem to move from the integral volume to the flux across the boundary of the elemental volume. In this way, changes in the cross-section averaged fluxes along the axial coordinate are obtained, as reported in Table 5.

It is worth noting that no approximation is introduced in the above equations, but we simply give up a complete description of the composition and temperature profiles, in favor of values averaged over the cross-section. Note that this system of Equations (23b–23d) is not closed unless assumptions are made on the average of the terms involving the product of the dependent variables and/or their partial derivatives. The simplest closure approximation is to assume that the average of the product can be factorized into the product of the cross-section averaged quantities. Nevertheless this approximation leads to some degree of error. Indeed, the term $d\langle \rho v_{z}c_{p}T\rangle /dz$ is usually written as $Fc_{p}dT/dz$, which does not represent an additional simplification if the cup-average quantity is considered, and $\langle r_{j}\rangle$ is usually evaluated as the reaction rate at the values of composition and temperature averaged over the cross-section, rather than as the average of the local rates of reaction. For this term, large errors can result if significant temperature gradients are present in the radial direction, due to the strong dependence of the reaction rates on temperature. In fact, even under isothermal conditions, the error on the rate of reaction can be significant when the reaction rate expression is strongly non linear with respect to the components’ concentrations. For example, using the data from the concentration profiles reported in Figure 4 of [59] with the kinetic expression of Wei and Iglesia [70], it can be found that the reaction rate evaluated at the average concentration is about two times higher than the average reaction rate, along the entire length of the reactor. This is an example of the hidden dangers of writing a simplified model, which, if done without the necessary attention, may lead to the introduction of greater inaccuracies than those expected. At the same time, in conditions in which the reaction rate is sufficiently high for equilibrium conditions to be reached locally in every point of the reactor, an error of this kind may not have a significant impact on the the final choice of design parameters or the evaluation of reactor performance.

Finally, 1D models are often further simplified by neglecting axial dispersion terms in the mass and heat balances; in this case, the classical plug flow model is recovered. Furthermore, in these models the evaluation of the axial velocity profile is no longer derived from the equation of continuity, but it is rather placed in relation to pressure drops along the reactor.

## 6. 2D Models

**Figure 3:**
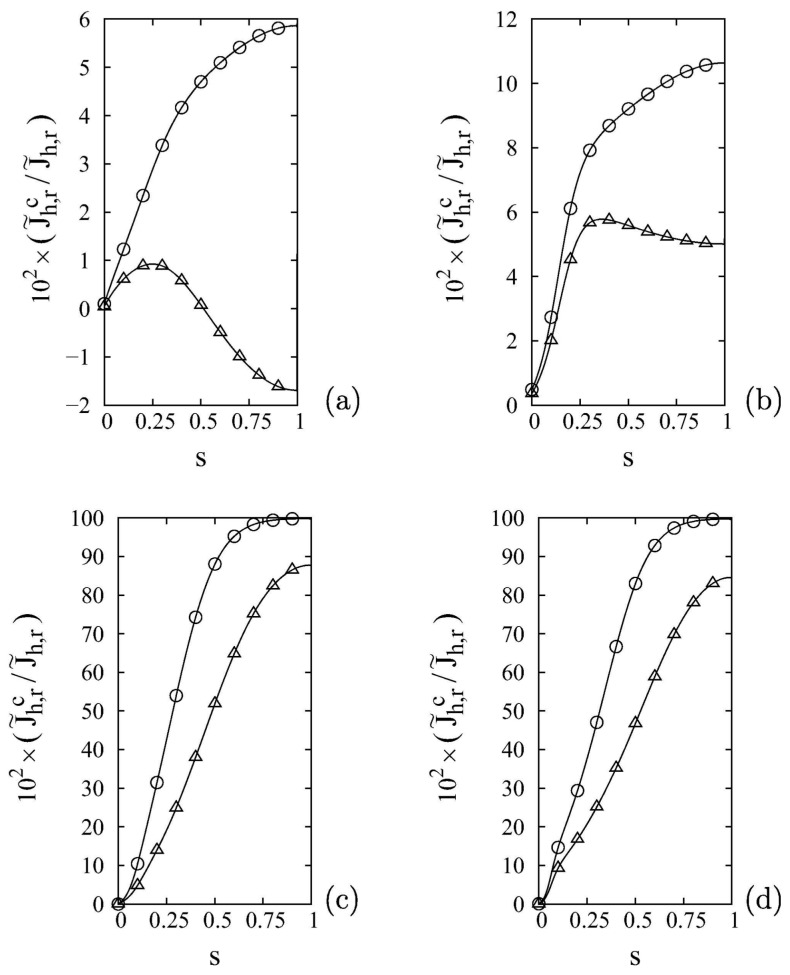
Ratio of the convective to the total radial component of the hydrogen flux at one third (circles) and two thirds (triangles) of the reactor length at fixed membrane permeability and reaction rate as a function of the dimensionless distance from the membrane, *s*, for values of the molecular Peclet number and pressure of (**a**) Pe=10, P=1 atm (**b**) Pe=10, P=10 atm (**c**) Pe=100, P=1 atm (**d**) Pe=190, P=10 atm. Reprinted from [59] with permission from Elsevier.

The first question that should arise when implementing a 2D model is whether it is sufficient to consider the axial velocity component, $v_{z}$, to describe the system. In principle, the presence of a permeable membrane on one of the reactor walls leads to a non-zero radial velocity component, $v_{r}$. The profile of such a velocity component may be non-trivial if one considers that the selective permeation of a component with molar mass significantly different from that of the other species, as is the case of hydrogen, causes the establishment of radial concentration (and therefore density) gradients, which, in turn, affect the behavior of $v_{r}$. In several works, the radial velocity component has been neglected without a preliminary order of magnitude analysis [71,72,73,74]; however, other works do take into account [10,21,57] the presence of radial convection. Indeed, it has been shown that in conditions of high membrane permeability or low mass dispersion, both of which favor the formation of steep radial concentration gradients, the value of $v_{r}$ is not negligible and by failing to consider it one risks making significant errors not only in the prediction of local concentration profiles but also of macroscopic quantities such as permeate flow rate and recovery [54,59,75]. Naturally, an accurate description of the system must go through the correct evaluation of the effective radial dispersion, which generally depends on the molecular diffusivity, the characteristic dimensions of the reactor and of the packing material, and the gas velocity. The radial effective dispersion in packed beds has been accounted for, through different correlations, by several groups [19,54,56,59,64]. An explicit analysis of the importance of the radial convective hydrogen flux compared to the radial dispersive flux in a membrane reactor under different operating conditions has been previously reported in [54,59] and is summarized by Figure 3. The figure shows that at low values of the Péclet number, Pe=U/(RD), the radial convective flux is only a few percentage points of the total radial flux, whereas at higher values of Pe, convection provides the major contribution to the radial transport of hydrogen. This result is an example of the fact that developing models with greater degrees of complexity allows their use over a wide variety of operating conditions. This, however, is done at the expense of the computational cost, which would not be justified should one be interested only in studying the behavior of the system in a limited range of conditions, for example at low values of Pe.

When neglecting the radial component of the velocity, dispersion is considered as the only mass transport mechanism in the radial direction. In this case the mass balance equation becomes

$$\rho \frac{\partial \omega_{i}}{\partial t}+\rho v_{z}\frac{\partial \omega_{i}}{\partial z}=\rho D_{e,z}\frac{\partial^{2}\omega_{i}}{\partial z^{2}}+\rho D_{e,r}\frac{1}{r}\frac{\partial }{\partial r}\left(r\frac{\partial \omega_{i}}{\partial r}\right)+M_{i}\sum_{j}\alpha_{i,j}r_{j}$$

with the following radial boundary conditions:

(24) $${\left.\left(-\rho D_{e,r}\frac{\partial \omega_{i}}{\partial r}\right)\right|}_{r=R_{1}}\cdot n_{1}=0\;\;\;\;\mathrm{for}\;i\neq H$$

(25) $${\left.\left(-\rho D_{e,r}\frac{\partial \omega_{h}}{\partial r}\right)\right|}_{r=R_{1}}\cdot n_{1}=J_{h}^{m}$$

(26) $${\left.\left(-\rho D_{e,r}\frac{\partial \omega_{i}}{\partial r}\right)\right|}_{r=R_{2}}\cdot n_{2}=0$$

where $R_{1}$ and $R_{2}$ indicate the membrane and impermeable wall radii, respectively, and $n_{1}$ and $n_{2}$ are local unit vectors normal to the surface and oriented outward the reaction volume.

In a similar way, a 2D description of temperature profile is usually written as

(27a) $${\left(\rho c_{p}\right)}_{bed}\frac{\partial T}{\partial t}+{\left(\rho c_{p}\right)}_{gas}v_{z}\frac{\partial T}{\partial z}=k_{e,z}\frac{\partial^{2}T}{\partial z^{2}}+k_{e,r}\frac{1}{r}\frac{\partial }{\partial r}\left(r\frac{\partial T}{\partial r}\right)+\sum_{j}r_{j}\left(-\Delta H_{j}\right)$$

The radial boundary conditions are

(27b) $${\left.\left(-k_{e,r}\frac{\partial T}{\partial r}\right)\right|}_{r=R_{1}}\cdot n_{1}={\dot{Q}}_{permeate}$$

(27c) $${\left.\left(-k_{e,r}\frac{\partial T}{\partial r}\right)\right|}_{r=R_{2}}\cdot n_{2}={\dot{Q}}_{wall}$$

where, as before, $R_{1}$ and $R_{2}$ indicate the membrane and impermeable wall radii, respectively, and $n_{1}$ and $n_{2}$ are local unit vectors normal to the surface and oriented outward the reaction volume.

## 7. Constitutive Equations

Whatever the level of complexity of the chosen model, constitutive equations for (i) reaction rates, (ii) hydrogen permeating flux and (iii) thermal flux through the reactor wall are required. Here we review shortly the equations used.

### 7.1. Reaction Rate Expressions

In this section we review reaction rate expressions for the steam reforming reactions that occur when using the three most common feeds, namely methane, ethanol and methanol. In any case, several reactions occur, also depending on the operating conditions.

#### 7.1.1. Methane Steam Reforming

The reactions that may take place during methane steam reforming are summarized in Table 6. It can easily be proved from thermodynamic considerations that only three of the seven reactions reported above are independent. Methane steam reforming in membrane reactors is usually carried out at temperatures of 700–900 K. Under these conditions, the formation of coke has been shown to be negligible, allowing to exclude reactions (28e)–(28g) and reducing the number of independent reactions to 2. The most commonly considered reactions are steam reforming (SR, Equation (28a)) and water-gas shift (WGS, Equation (28b)) (see, e.g., [4,76]). Furthermore, given the lower temperatures employed in membrane steam reformers compared to traditional reactors, it has been experimentally shown that the WGS reaction reaches completion [77], making it possible to describe the system simply through the overall steam reforming (OSR) reaction (Equation (28d)).

In any case, as for the process kinetics, the most commonly used [5,10,57,78,79,80] reaction rate expressions for SR, WGS, and OSR are those derived by Xu and Froment [81] on a Ni/MgAl_2_O_4_ catalyst, which read, respectively

(29a) $$r_{SR}=\frac{\frac{k_{SR}p_{CH_{4}}p_{H_{2}O}}{p_{H_{2}}^{2.5}}\left(1-\eta_{SR}\right)}{{\left(DEN\right)}^{2}}$$

(29b) $$r_{WGS}=\frac{\frac{k_{WGS}p_{CO}p_{H_{2}O}}{p_{H_{2}}}\left(1-\eta_{WGS}\right)}{{\left(DEN\right)}^{2}}$$

(29c) $$r_{OSR}=\frac{\frac{k_{OSR}p_{CH_{4}}p_{H_{2}O}^{2}}{p_{H_{2}}^{3.5}}\left(1-\eta_{OSR}\right)}{{\left(DEN\right)}^{2}}$$

where

(29d) $$DEN=1+K_{CO}p_{CO}+K_{H_{2}}p_{H_{2}}+K_{CH_{4}}p_{CH_{4}}+K_{H_{2}O}p_{H_{2}O}/p_{H_{2}}$$

In the equations above, $k_{1}$, $k_{2}$, and $k_{3}$ are the SR, WGS, and OSR reaction rate constants; $K_{1}$, $K_{2}$, and $K_{3}$ are the corresponding equilibrium constants; and the $K_{i}$s appearing in Equation (29d) are the adsorption equilibrium constants for the different species on the catalysts particles; $1-\eta_{i}$ is the distance from the equilibrium of the i−th reaction (for example, for the SR reaction $1-\eta_{SR}=1-p_{H2}^{3}p_{CO}/\left(K_{1}p_{CH4}p_{H2O}\right)$). When equilibrium conditions are reached, $\eta_{i}=1$ and the reaction stops. Similar expressions have been proposed by Hou and Hughes [82] and by Soria et al. [83]. The expressions proposed by the latter differ from those of Xu and Froment only for the absence of the denominator accounting for adsorption of the different species on the catalyst

(30a) $$r_{SR}=\frac{k_{SR}p_{CH_{4}}p_{H_{2}O}}{p_{H_{2}}^{2.5}}\left(1-\eta_{SR}\right)$$

(30b) $$r_{WGS}=\frac{k_{WGS}p_{CO}p_{H_{2}O}}{p_{H_{2}}}\left(1-\eta_{WGS}\right)$$

(30c) $$r_{OSR}=\frac{k_{OSR}p_{CH_{4}}p_{H_{2}O}^{2}}{p_{H_{2}}^{3.5}}\left(1-\eta_{OSR}\right)$$

The main drawback of these expressions is represented by the fact that one obtains an infinite reaction rate when hydrogen is not present in the gas mixture, as is common in the inlet section.

Other works [59,84] have made use of the considerably simpler expression proposed by Wei and Iglesia [70], according to which the direct SR reaction rate is proportional to the methane partial pressure and the methane consumption rate is therefore written as

(31) $$r_{CH_{4}}=k_{SR}p_{CH_{4}}\left(1-\eta_{SR}\right)$$

The same expression can be used to describe the total steam reforming reaction, with the obvious changes to the term η.

Simple expressions for the SR and WGS reactions have also been proposed by Numagauchi and Kikuchi [85]. A thorough discussion and review of the description of the rate of reaction for low temperature methane steam reforming may be found in [86], whereas reaction rate expressions proposed for methane steam reforming in a wider temperature range on Ni-based catalysts are summarized in [87]. It is worth mentioning that several authors have reported that in commonly used operating conditions and with the catalysts available the reaction rate is often high enough as to make it possible to consider local equilibrium in every point of the reactor [4,5,12,55].

#### 7.1.2. Ethanol Steam Reforming

When carrying out the ethanol steam reforming, in addition to the desired ethanol reforming (32a) and overall reforming (32b) reactions, all the reactions reported in Table 6 and Table 7 could also take place [88]. The high number of reactions testifies to the difficulty of directing selectivity towards the desired products when carrying out ethanol steam reforming.

Tests carried out on a CeO_2_-supported Pt/Ni catalyst, for temperatures ranging between 250 and 600 °C, a GHSV of 15,000 h^−1^ and water to ethanol ratio of 3 [88], indicated that the reactions taking place are ethanol dehydration (EDH), acetaldehyde decomposition (AD), acetaldehyde steam reforming (ASR), ethanol decomposition (ED), and CO_2_ methanation (M) (reactions 32c–32g), along with the WGS reaction (28b). The corresponding rate expressions proposed are

(33a) $$r_{EDH}=k_{EDH}p_{EtOH}\left(1-\eta_{EDH}\right)$$

(33b) $$r_{AD}=k_{AD}p_{Acet}\left(1-\eta_{AD}\right)$$

(33c) $$r_{ASR}=k_{ASR}p_{Acet}p_{H_{2}O}\left(1-\eta_{ASR}\right)$$

(33d) $$r_{ED}=k_{ED}p_{EtOH}\left(1-\eta_{ED}\right)$$

(33e) $$r_{M}=k_{M}p_{CO_{2}}p_{H_{2}O}^{4}\left(1-\eta_{M}\right)$$

(33f) $$r_{WGS}=k_{WGS}\frac{\left(K_{CO_{2}}K_{H_{2}O}\right)p_{CO}p_{H_{2}O}\left(1-\eta_{WGS}\right)}{{\left(1+K_{CO}p_{CO}K_{H_{2}O}p_{H_{2}O}+K_{CO_{2}}p_{CO_{2}}\right)}^{2}}$$

where $\left(1-\eta \right)$ has the same meaning of a distance from equilibrium as defined earlier.

If no acetaldehyde formation takes place, reactions (32c) and (32d) may be combined into the modified ethanol decomposition reaction

(34) $$C_{2}H_{5}\mathrm{OH}⇌H_{2}+{\mathrm{CH}}_{4}+\mathrm{CO}$$

and reaction (32e) may be neglected. Of the five reactions remaining, only three are linearly independent, and the system may be studied in terms of the modified ethanol decomposition decomposition to H_2_, CO and CH_4_ (34), WGS (28b), and CO_2_ methanation (32g) [23].

The same set of reactions has been used to describe ethanol steam reforming over Ni- and Co-based catalysts [25,26]. Gallucci et al. [26], used the reaction rate expressions developed by Sahoo et al. [89], who proposed a surface reaction mechanistic kinetic model using a Langmuir–Hinshelwood approach based on both literature and product distribution obtained in the course of their study.

A review of the catalysts proposed for ethanol steam reforming, the reaction pathway on each catalyst, and the performance in terms of activity and selectivity towards hydrogen production is reported in [90].

#### 7.1.3. Methanol Steam Reforming

In this case, the main reactions that take place are methanol steam reforming

(35a) $${\mathrm{CH}}_{3}\mathrm{OH}+H_{2}O⇌{\mathrm{CO}}_{2}+3H_{2}$$

methanol decomposition

(35b) $${\mathrm{CH}}_{3}\mathrm{OH}⇌2H_{2}+\mathrm{CO}$$

and WGS.

In some cases, authors claimed that the amount of carbon monoxide produced when carrying out the steam reforming of methanol was so low that it could only be produced by the reverse WGS reaction, thus excluding methanol decomposition [91,92].

Power-law models for the rate of the direct methanol steam reforming reactions have been developed by some groups to describe their experimental data, including Lee et al. [91] and Samms and Savinell [93]. Other groups proposed instead Langmuir–Hinshelwood mechanisms based on different reaction mechanisms. Tesser et al. [94] considered a negative effect of water and hydrogen on the reaction rate and proposed the following expression for the rate of the direct methanol steam reforming reaction

(36) $$r_{MeOH}=\frac{k_{MeOH}K_{CH_{3}OH}\;p_{CH_{3}OH}}{1+K_{CH_{3}OH}+K_{H_{2}O}\;p_{H_{2}O}+K_{H_{2}}\;p_{H_{2}}}$$

Several authors reported the existence of two distinct types of active sites on Cu/ZnO/Al_2_O_3_ catalysts [91,92]. Among these, the kinetic model developed by Peppley et al. [95] was based on the following underlying ideas: (i) hydrogen and the oxygen-containing species do not compete for the same catalyst active sites, (ii) the methanol steam reforming and water gas shift reactions take place on different active sites than the methanol decomposition reaction, (iii) the methanol SR and decomposition reactions are limited by the dehydrogenation of adsorbed methoxy groups, and (iv) the rate of the WGS reaction is limited by the formation of an intermediate species. The resulting reaction rate expressions for methanol steam reforming, methanol decomposition, and water gas shift, which are commonly used when modeling membrane reactors, are, respectively

(37a) $$r_{1}=\frac{k_{1}K_{CH_{3}O}^{*}\left(p_{CH_{3}OH/p_{H_{2}}^{0.5}}\right)\left(1-p_{H_{2}}^{3}p_{CO_{2}}/K_{1}p_{CH_{3}OH}p_{H_{2}O}\right)C_{s1}C_{s1a}}{DEN}$$

(37b) $$r_{2}=\frac{k_{2}K_{CH_{3}O}^{*}\left(p_{CH_{3}OH/p_{H_{2}}^{0.5}}\right)\left(1-p_{H_{2}}^{2}p_{CO}/K_{2}p_{CH_{3}OH}\right)C_{s2}C_{s2a}}{DEN}$$

(37c) $$r_{3}=\frac{k_{3}K_{OH}^{*}\left(p_{CO}p_{H_{2}O}/p_{H_{2}}^{0.5}\right)\left(1-p_{H_{2}}p_{CO_{2}}/K_{3}p_{CO}p_{H_{2}O}\right)C_{s1}^{2}}{DEN}$$

where

(37d) $$\begin{array}{c} DEN=1+K_{CH_{3}O}^{*}\left(p_{CH_{3}OH}/p_{H_{2}}^{0.5}\right)+K_{HCOO}^{*}p_{CO_{2}}p_{H_{2}}^{0.5}+\\ K_{OH}^{*}\left(p_{H_{2}O}/p_{H_{2}}^{0.5}\right)\left(1+K_{H}^{{*}^{0.5}}p_{H_{2}}^{0.5}\right) \end{array}$$

$C_{s1}$, $C_{s1a}$, $C_{s2}$, $C_{s2a}$ are the concentrations of catalyst sites, and $K_{i}^{*}$ are the adsorption coefficients of the i-th species. These expressions have been used in several works on methanol SR in membrane reactors [15,19,21,96].

An overview of proposed models of the reaction mechanism and rate expressions of methanol steam reforming along with a comparison of experimental data against different expressions proposed in literature is reported in the work of Sa et al. [97].

### 7.2. Hydrogen Permeating Flux

As mentioned in the introduction, membrane reactors for hydrogen production make use of Pd-based membranes, which present a virtually infinite selectivity towards hydrogen and high hydrogen permeability. The permeating flux through these membranes is usually described by Sieverts’ law and depends on the difference between the square root of the partial pressures of hydrogen in the retentate $\left(p_{H_{2}}^{r}\right)$ and permeate ($p_{H_{2}}^{p}$) sides, in contact with the membrane

(38) $$J_{h}^{m}=\phi \left(\sqrt{p_{H_{2}}^{r}}-\sqrt{p_{H_{2}}^{p}}\right)$$

where $J_{h}^{m}$ is the hydrogen flux across the membrane and ϕ is the membrane permeance. The value of the membrane permeance depends on factors including its thickness, composition, and fabrication procedures. Discussions on and values of the permeance of hydrogen-permeable membranes are reported in [98,99,100].

Equation (38) is usually employed to evaluate the permeating flow both in tests with pure hydrogen and with hydrogen-containing gas mixtures; although in the last case it has been noticed that a significant reduction in the apparent permeance value must be considered in order to adequately describe the experimental results [5,20,23]. Indeed, such a reduction in the hydrogen flow may be due to at least two different phenomena: (i) a competitive adsorption of some components on the membrane surface, particularly CO [20,101,102,103,104,105,106,107,108,109,110,111,112]; (ii) the presence of different transport resistances in the system, i.e., concentration polarization effects.

To account for inhibition due to competitive adsorption on the membrane’s surface, several expressions have been proposed in literature [20,37,113]

(39a) $$J_{h}^{m}=\phi \frac{1}{1+\sqrt{K_{H}p_{H}^{r}}+\sum_{i}K_{i}p_{i}^{r}}\left(\sqrt{p_{H}^{r}}-\sqrt{p_{H}^{p}}\right)$$

(39b) $$J_{h}^{m}=\phi \left(\frac{\sqrt{p_{H}^{r}}}{1+\sqrt{K_{H}p_{H}^{r}}+\sum_{i}K_{i}p_{i}^{r}}-\sqrt{p_{H}^{p}}\right)$$

(39c) $$J_{h}^{m}=\phi \frac{1+\sqrt{K_{H}p_{H}^{r}}}{1+\sqrt{K_{H}p_{H}^{r}}+\sum_{i}{\left(K_{i}p_{i}^{r}\right)}^{i}}\left(\sqrt{p_{H}^{r}}-\sqrt{p_{H}^{p}}\right)$$

where the subscripts *H* and *i* are used to indicate hydrogen and the the i−th inhibiting reactant, respectively, and *K* is the adsorption constant of each component.

Equation (39a) is based on the assumption that the driving force for diffusion is the gradient in hydrogen coverage and that this quantity is described by the Langmuir isotherm; however, it has been criticized [114] because it shows the same inhibition effect on both the retentate and permeate sides of the membrane, even though the inhibitor (reactant) is only present in the retentate side. Equation (39b) corrects this aspect, but if inhibition is strong it predicts hydrogen transport in the wrong direction. Finally, Equation (39c) is an empirical expression that matches Sieverts’ law in the absence of inhibition. Barbieri et al. [115], on the other hand, modified Sieverts’ law in the presence of CO as follows

(40) $$J_{h}^{m}=\phi \left(1-\alpha \left(T\right)\frac{K_{CO}p_{CO}}{1+K_{CO}p_{CO}}\right)\left(\sqrt{p_{H}^{r}}-\sqrt{p_{H}^{p}}\right)$$

where α(T) is a Langmuir affinity parameter.

A new correlation was also proposed in [116], in which the resistance to the transport of atomic hydrogen from the membrane surface to the first layer of the membrane bulk was accounted for. The resulting expression was

(41) $$J_{H}=\frac{D}{L}\left(\psi -\psi^{p}\right)$$

where D is the diffusivity of atomic hydrogen in the membrane, *L* is the membrane thickness, and ψ and $\psi^{p}$ represent the H occupancy in the first layer of the bulk of the membrane immediately after the surface exposed in the retentate side, and in the last layer immediately before the surface exposed to the permeate side, respectively and the correlations through which they are evalauted are presented in [116]. The same work also proposes a simplified expression, valid under commonly employed reaction conditions, in which the inhibition factor, Θ, defined as the ratio between the flux across the membrane in the presence of inhibition and in the absence of inhibition, may be evaluated as

(42) $$\Theta =\frac{1}{1+\frac{1}{6n}\left(1+K_{CO}p_{CO}\right)}$$

where *n* is the thickness of the membrane, expressed in terms of number of Pd atoms.

In addition to determining the most accurate expression to describe flux reduction due to competitive adsorption, accurate values of the adsorption equilibrium constants need to be evaluated. According to the kinetic theory, the adsorption equilibrium constant may be estimated as

(43) $$K_{eq}=\frac{1}{C_{t}\frac{k_{B}T}{h}\sqrt{2\pi mk_{B}T}}\exp\left(\frac{-\Delta E_{ads}}{k_{B}T}\right)\exp\left(\frac{\Delta S}{k_{B}}\right)$$

where $C_{t}$ is the density of sites, taken to be $10^{19}\;m^{-2}$, *m* is the mass of the adsorbed molecule, *T* is the temperature in *K*, $k_{B}$ and *h* are the Boltzmann and Planck constants, respectively, $E_{ads}$ is the energy of the adsorption reaction and $\Delta S_{ads}$ is the entropy change. In many cases, adsorption is considered to be non-activated, and the issue of finding the adsorption equilibrium constant is reduced to the determination of the adsorption energy ($\Delta E_{ads}$), the change in entropy ($\Delta S_{ads}$) and the number of sites occupied by each molecule. Several attempts have been made to study the adsorption of CO and H_2_ through first principles [102,117,118,119,120].

Empirical correlations for the adsorption constants of the inhibiting components have been proposed by Israni and Harold [20], who studied the effects of the presence of the components involved in methanol steam reforming on the hydrogen flux across a Pd/Ag membrane. Of the components present during this process, CO was found to have the most significant inhibiting effect. The expressions of the adsorption equilibrium constants as a function of temperature for the various components were determined and used by the authors in an expression similar to that of Equation (39c). The expressions obtained by the authors for H_2_ and CO are

(44) $$K_{H}=3.33\times 10^{-10}exp\left(\frac{58462(J/mol)}{RT}\right)\left[\frac{1}{Pa}\right]$$

(45) $$K_{CO}=6.38\times 10^{-11}exp\left(\frac{88423(J/mol)}{RT}\right)\left[\frac{1}{Pa}\right]$$

As for the presence of different transport mechanisms which govern the permeating flux, e.g., the mass transport in the packed bed or in the permeate side, it is worth considering that in a 2D model these additional resistances are accounted for simply by using the hydrogen partial pressure at the interface with the membrane in Sieverts’ law. Different approaches must be instead used in simplified 1D models. In particular, the presence of the additional resistances has been accounted for by empirically changing the exponent of pressure in Sieverts’ law [121]

(46) $$J_{h}^{m}=\phi \left({\left(p_{H_{2}}^{r}\right)}^{n}-{\left(p_{H_{2}}^{p}\right)}^{n}\right)$$

however, this approach gives no insight into the physical transport processes, and limits the applicability of the models developed to the specific equipment geometry and operating conditions used for estimating the power-law exponent. In fact, it was found that modifying Sieverts’ law may also serve to account for membrane defects [122]. A different approach consists in evaluating a mass transport coefficient in the gaseous phase to describe the concentration boundary layer of the permeating component between the bulk of the gas and the membrane surface [123,124,125]. In particular, Catalano et al. [125] modify the mass transfer coefficient in order to account for variations in velocity profiles arising in presence of high permeating flux in an empty membrane separator. An interesting alternative has been proposed by Nekhamkina and Sheintuch [69], who developed an approximate model to simulate the trans-membrane hydrogen flux in an empty membrane separator. The hydrodynamic and diffusion problems were separated by constructing an approximate hydrodynamic field under the assumption of constant density. A dimensionless parameter Γ, which represents the ratio between the diffusive and permeating fluxes, was introduced, defining the range of operating conditions for which the effects of concentration polarization may be neglected. In any case, it has been shown that the apparent permeance can be considerably smaller than the one measured in pure hydrogen, even after accounting for dilution, concentration gradients, and inhibition. The occurrence of hydrogen-consuming reactions on the surface of the membrane has also been proposed as a possible reason for the drop in the observed hydrogen flux [5,32,112] and may strongly affect the reactor performance.

### 7.3. Heat Exchange with the Reactor Wall and Permeate

When writing the energy equation of change for the retentate side, heat exchange with the reactor wall and the permeate side should be also accounted for. With regards to the exchange with the reactor wall, two approaches are generally adopted. The first consists in considering a wall maintained at constant temperature through an external heating system and, consequently, the description of heat transfer between the wall and catalyst in terms of a heat transfer coefficient [5,23,28,80]

(47) $${\dot{Q}}_{wall}=U_{w}\left(T_{w}-T\right)$$

where $U_{w}$ is the heat transfer coefficient between the wall and the catalyst and $T_{w}$ is the wall temperature.

In other instances radial temperature variations within the wall are accounted for and $T_{w}$ is therefore no longer a known problem parameter, but rather a quantity that depends on the relative importance of heat transfer between the wall and both the packed bed and the furnace (or other heating system) [21]. The term ${\dot{Q}}_{wall}$ appearing the energy balance equation of the retentate remains unchanged.

Although heat transfer with the permeate is often neglected [5,21,80], in principle two contributions are present, one associated to heat conduction across the membrane, and the other to the enthalpy carried by hydrogen when permeating across the membrane. If both factors are considered one obtains [25,28]

(48) $${\dot{Q}}_{permeate}=U_{m}\left(T-T^{p}\right)+J_{h}^{m}\left(c_{p,H_{2}}^{r}T-c_{p,H_{2}}^{p}T^{p}\right)$$

where $U_{m}$ is the heat transfer coefficient between the catalyst and the permeate, and the superscript *p* identifies variables that are defined in the permeate.

Although a detailed discussion of expressions for the heat transfer coefficient is beyond the scope of the present work, a brief summary of correlations proposed in the literature is reported. With regards to transport in packed beds, the general expressions proposed for $U_{w}$ and $U_{m}$ are similar. The heat transfer coefficients are determined from the Nusselt number, which in turn may depend on the Prandtl and particle Reynolds numbers [126,127,128,129,130,131]

(49) $$Nu_{w}=a+bRe^{\alpha }Pr^{\beta }$$

where the coefficients depend on the gas velocity, the characteristic dimension of the reactor, and on the size and material of the catalyst pellets.

The corresponding correlations for the heat transfer in solid foams proposed in the literature are less numerous and more diverse. This is partly due to the variability in cell size and strut diameter within the same foam and between foams produced by different manufacturers. To overcome this limitation, Busse et al. [132] proposed the use of periodic open cellular structure, in which the representative unit cell is repeated in each spatial direction, resulting in a highly regular structure. The resulting wall-to-bed Nusselt number was determined to be

(50) $$Nu_{w}=4.51+0.029Re_{lc}^{0.8}$$

where $Re_{lc}$ is the Reynolds number based on the characteristic cell size, $l_{c}$.

This expression is similar to those used to described heat transfer in packed beds and has been derived by the authors on the basis of the correlation proposed by Bianchi et al. [133], who studied heat transport in metallic foams

(51) $$Nu_{w}=7.18+0.029Re_{lc}^{0.8}$$

Indeed, the two expressions differ only in the value of the first term, which accounts for heat transfer by conduction.

Dietrich [134] proposed instead the following correlation for heat transfer in ceramic foams

(52) $$Nu_{w}=0.31\;Hg^{1/3}Pr^{1/3}$$

where Hg is the Hagen number, defined as

(53) $$Hg=\frac{\Delta p}{\Delta L}\frac{d_{h}^{3}}{\rho_{f}\nu_{f}^{2}}$$

where Δp/ΔL are the pressure drops per unit length of the reactor, $d_{h}$ is the hydraulic diameter of the foams, $\rho_{f}$ is the density of the fluid, and $\nu_{f}$ its kinematic viscosity. The expression was derived from the Generalized Léveque Equation, which relates the heat transfer coefficient to pressure drop data. Its validity was confirmed experimentally by a different group [135], who also highlighted the absence of a universal expression for the evaluation of the foam’s specific surface area, required to determine the hydraulic diameter.

## 8. Some Literature 1D Models

In this section we present an overview of selected 1D models proposed in literature, describing in some detail the operating conditions, simplifying assumptions and model equations.

**Figure 4:**
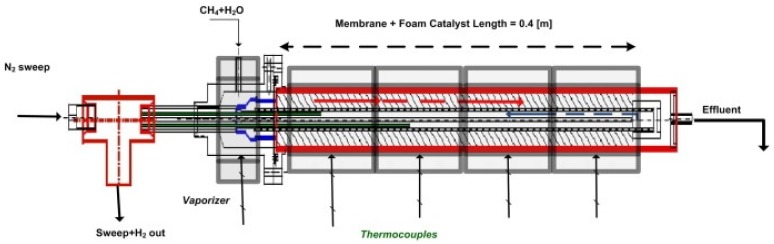
Detailed drawing of the reactor used by Patrascu and Sheintuch [5]. Reprinted from the original reference with permission from Elsevier.

Patrascu and Sheintuch [5] developed a 1D transient model for methane steam reforming and compared the calculated results with those obtained from an experimental campaign. The reactor geometry consisted of a shell and tube configuration, in which the catalyst, supported on a solid foam, is placed in the annular volume and the membrane is supported on the outer wall of the innermost tube. The permeate flows in the inner cylindrical volume in counter-current mode with respect to the gas flow in the retentate side. A detailed drawing of the reactor is shown in Figure 4.

The reactions considered were methane steam reforming (Equation (28a)), water-gas shift (Equation (28b)), and overall steam reforming (Equation (28d)). The reactor is heated from the external wall, which is assumed at constant temperature. Reaction rates were described through the expressions proposed by Xu and Froment [81].

The model developed by the authors accounted for heat and mass balance in both the retentate and permeate sides, neglecting the pressure losses; axial dispersion was accounted for and the Danckwerts boundary conditions at the reactor inlet were used both in mass and energy equations of change. Constant mass dispersion coefficient and effective thermal conductivity were assumed. The permeate side was always left at atmospheric pressure; when no sweep gas was used, the hydrogen pressure in the permeate was set to 1 bar and if the partial pressure of hydrogen in the retentate side dropped below 1 bar the membrane permeability was set to zero to avoid the unrealistic back-permeation of hydrogen. The heat capacities of permeate and sweep gas were neglected. Most of the model was predictive, whereas the inhibition factor was adjusted to better fit the experimental results while gaining more insight into the physical phenomena determining the performance of the system. In fact, the approach followed to describe hydrogen flow through the membrane is particularly worthy of attention.

A corrected form of Sieverts’ law was proposed as follows

(54) $$J_{h}^{m}=\Theta \phi \left(\sqrt{p_{H_{2}}^{r}}-\sqrt{p_{H_{2}}^{p}}\right)$$

where the inhibition factor Θ is introduced. In a preliminary analysis the authors show that concentration polarization is not negligible, but cannot predict the observed reduction in the hydrogen flow. Therefore, the authors considered three different methods for the evaluation of Θ: (i) treating is as an empirical parameter to be calibrated at a constant value from experimental data at specific working conditions, (ii) evaluating it using the empirical correlations proposed in [20] to account for CO competitive adsorption and Equation (39c), or (iii) evaluating as in the previous case while considering the inhibiting effect of methane to be equal to that of CO. Indeed, both the assumption of a constant value of Θ (0.18) and its evaluation under the assumption of inhibition by CO only did not allow an accurate description of the experimental data. Specifically, it was not possible to account for the observed changes in permeating flux and methane conversion with the increase in operating pressure. The last method, finally used in the model, derived form the idea that methane could react with water on the membrane surface, forming CO. The inhibition factor was therefore evaluated through the expression previously derived by Israni and Harold [20], while assuming the same inhibition effect for CO and CH_4_ and neglecting the inhibition by all other components.

Next, we move on to consider the work by Pieterse et al. [84], which is particularly interesting because it provides significant insight through an extremely simple model. In fact, the authors’ objective in the first part of their work was to evaluate the catalytic activity required in a membrane reactor to avoid the reaction from becoming the limiting step. To this aim, the authors use a very simple 1D model to predict the gas composition in an isothermal membrane reformer fed with a gas mixture from a pre-reforming unit without membrane. Such a configuration is very often considered, due to the high cost of the membrane. The envisaged reactor configuration is one in which the sweep gas flows in the central cylindrical volume and the catalyst is placed in the outer annular volume, such as the one depicted in Figure 1b.

The model developed to answer this question is a simple plug-flow isothermal model. The total and partial mass transport equations are, respectively

(55) $$\frac{du}{dz}=\frac{\rho_{b}}{c_{t}}\sum_{I,i}r_{I,i}-\frac{1}{d_{b}c_{t}}\sum_{i}J_{i}$$

(56) $$\frac{dc_{i}}{dz}=-\frac{c_{i}}{u}\frac{du}{dz}+\frac{\rho_{b}\sum_{I}r_{Ii}}{u}-\frac{J_{i}}{d_{b}u}$$

where $c_{i}$ is the concentration of the i−th component (CH_4_, H_2_O, CO, CO_2_, H_2_), *u* is the superficial gas velocity, $\rho_{b}$ is the bed density, $r_{I,i}$ is the net reaction rate of the i−th component in the I−th reaction, $J_{i}$ is the cross-membrane flux of the i−th component, $d_{b}$ the catalyst bed width, and $c_{t}$ the total concentration.

The authors explicitly claim that, in light of the objective for which their model was developed, all radial resistances to mass transport, with the exception of the membrane permeance were neglected. In this manner, the permeating flux of hydrogen is overestimated and a conservative value of the catalytic activity required to sustain hydrogen permeation is obtained. The hydrogen flow through the membrane was evaluated by considering the driving force to be the difference between the hydrogen partial pressure in the retentate and permeate side. Different literature kinetic models were considered. The authors plotted the length-averaged value of η (i.e., proximity to chemical equilibrium), achieved by the catalyst as a function of the membrane permeance. For increasing values of the permeance, η reaches values significantly smaller than unity, meaning that the catalyst is no longer capable of maintaining equilibrium conditions and the capabilities of the membrane, which is the most expensive component of the reactor, are not fully exploited.

This work is an excellent example of an instance in which the simplifying assumptions have been tailored to the use to be made of the model and have been discussed exhaustively. Since the objective was to determine the minimum catalytic activity required to avoid the reaction from becoming the limiting phenomenon, a detailed description of the concentration profiles would have increased the computational cost, and the effort required to accurately determine the model parameters, without providing any meaningful advantage. In addition, the choice of neglecting the resistance to radial transport led to the determination of a conservative value of the catalytic activity, thereby allowing an extension of the results even to operating conditions that may differ slightly from those considered.

## 9. Some Literature 2D Models

In the following paragraphs, 2D models are reported in a similar fashion as done in the previous section for 1D models. We start with the steady-state, pseudo-homogeneous, non-isothermal model presented by Marin et al. [64] for methane reforming on Ru/SiO_2_ catalyst particles carried out in a shell and tube reactor. In the proposed configuration the membrane forms the wall of the inner tube, which contains the catalyst, and the permeate flows in the outer annular volume, which is heated externally.

Momentum transport was described through the Brinkman equation

(57) $$\nabla \cdot \left[-pI+\frac{\mu }{\varepsilon_{b}}\left(\nabla v+{\left(\nabla v\right)}^{T}\right)-\frac{2\mu }{3\varepsilon_{b}}\left(\nabla \cdot v\right)I\right]-\left(\frac{\mu }{\kappa_{D}}+\beta_{F}\left|v\right|\right)v=0$$

with the boundary conditions

(58) $${\left.v\right|}_{z=0}\cdot n=v_{z}^{in}$$

(59) $${\left.P\right|}_{z=L}=P_{out}$$

(60) $${\left.v\right|}_{r=r_{m}}\cdot n_{1}=v_{r}^{m}=\frac{D_{int}}{D_{R}}\frac{J_{m}}{c_{Gm}}$$

(61) $${\left.v=0\right|}_{r=0}\cdot n_{2}=0$$

where $J_{m}$ is the permeating hydrogen flux, described through Sieverts’ law, $c_{Gm}$ is the molar concentration of the gas in proximity of the membrane, and $D_{int}$ and $D_{R}$ are the internal diameter of the outer tube and the bed diameter, respectively.

The mass and energy balance equations accounted for the effects of convection, dispersion, and reaction, yielding for mass transport

(62) $$-v\cdot \nabla c_{i}+\nabla \cdot \left(D_{ie}\nabla c_{i}\right)+\left(1-\varepsilon_{b}\right)\rho_{s}\sum_{j=1}^{N_{r}}\nu_{ij}\eta_{j}r_{ij}=0$$

where $D_{ie}$ is the effective dispersion tensor of the *i-th* species

(63) $$D_{ie}=\left[\begin{array}{cc} D_{ier} & 0\\ 0 & D_{iez} \end{array}\right]$$

The values of the the effective dispersion coefficients were determined from literature correlations.

The reaction rate expressions used were those obtained in another work by some of the same authors [83], discussed in Section 7.1.1, and corrected through empirical correlations for the effectiveness factors, obtained by solving the particle mass and energy balance equations for different operating conditions within the ranges considered in the work.

The boundary conditions read

(64) $${\left.c_{i}=y_{i,in}c_{Gin}\right|}_{z=0}$$

(65) $${\left.\left(-D_{ie}\nabla c_{i}\right)\right|}_{z=L}\cdot n=0$$

(66) $${\left.\left(-D_{ie}\nabla c_{i}+\varepsilon_{b}c_{i}v\right)\right|}_{r=r_{m}}\cdot n_{1}=\frac{D_{int}}{D_{r}}J_{m}$$

(67) $${\left.\left(-D_{ie}\nabla c_{i}+\varepsilon_{b}c_{i}v\right)\right|}_{r=0}\cdot n_{2}=0$$

The energy balance equation is

(68) $$-\rho_{g}C_{pG}v\cdot \nabla T+\nabla \cdot \left(k_{e}\cdot \nabla T\right)-\left(1-\varepsilon_{b}\right)\rho_{s}\sum_{j=1}^{N_{R}}\delta H_{j}\eta_{j}r_{mj}=0$$

where $k_{e}$ is the effective heat conductivity tensor

(69) $$k_{e}=\left[\begin{array}{cc} k_{er} & 0\\ 0 & k_{ez} \end{array}\right]$$

and the boundary conditions are

(70) $${\left.T=T_{in}\right|}_{z=0}$$

(71) $${\left.t\left(-k_{e}\nabla T\right)\right|}_{z=L}\cdot n=0$$

(72) $${\left.\left(-k_{e}\nabla T\right)\right|}_{r=r_{m}}\cdot n_{1}=h_{mem}\left(T-T_{sh}\right)$$

(73) $${\left.\left(-k_{e}\nabla T+\varepsilon_{b}\rho_{G}C_{PG}Tv\right)\right|}_{r=0}\cdot n_{2}=0$$

where $T_{sh}$ is the temperature in the shell (permeate) and $h_{mem}$ is the heat transfer coefficient between the permeate and retentate sides.

Since hydrogen concentration and temperature in the permeate are considered to vary only in the axial direction, the following 1D model is proposed for the permeate

(74) $$\frac{dF_{H_{2}}}{dz}=\pi D_{int}J_{m}$$

(75) $$\frac{dT_{sh}}{dz}=\frac{\pi D_{ext}h_{oven}\left(T_{oven}-T_{sh}\right)-\pi D_{int}h_{mem}\left(T_{sh}-T\right)}{\left(F_{H_{2}}+F_{sweep}\right)C_{Psh}}$$

to be solved, respectively, with the boundary conditions

(76) $${\left.F_{H_{2}}\right|}_{z=0}=0$$

(77) $${\left.T_{sh}\right|}_{z=0}=T_{s}$$

where $D_{int}$ and $D_{ext}$ are the internal and external diameters of the outer tube, respectively; $h_{oven}$ is the heat transfer coefficient between the oven and the outer reactor wall, and $C_{Psh}$ is the specific heat of the gas in the permeate side.

In the same work the authors use a top-down approach to develop a simplified 1D model in which the Brinkman equation is substituted by the Darcy equation and the energy balance equation is modified by the introduction of a global heat transfer coefficient that accounts for heat transfer both between the packed bed and the permeate (shell) side and within the packed bed. The results of the two models are compared and significant discrepancies are found between the two, mainly due to the lower heat transfer rate from the reactor wall to the catalyst bed estimated by the 1D model. It should be noted that, since the model was used to describe a specific setup, an alternative approach may have been represented by an adjustment of the empirical heat transfer parameters.

**Figure 5:**
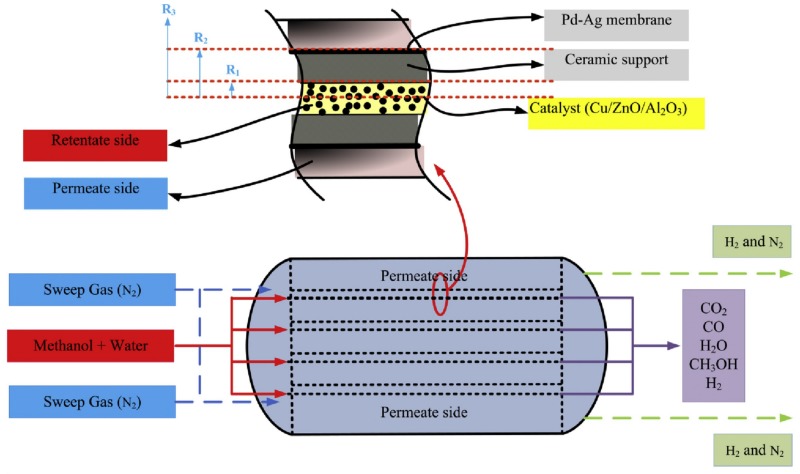
Scheme of the reactor modeled by Saidi et al. [19]. Reprinted from the original reference with permission from Elsevier.

Saidi [19] proposed a 2D model for the description of a methanol steam reforming reactor. The final configuration envisaged consists of 600 tubes containing the catalyst and placed in an exterior shell in which the permeate gas flows. The model is limited to a single tube, as shown in Figure 5.

The reactions considered are methanol steam reforming, water-gas shift, and methanol decomposition, and their rates are described through the expressions proposed by Peppley et al. [95]. The steady-state model is based on the following simplifying assumptions

- negligible pressure drops
- negligible axial dispersion
- negligible radial convective mass transfer
- negligible heat and mass transfer resistances between the gas and catalyst

The authors also include the balance equations in the membrane ceramic support. All variables and parameters referring to the ceramic support will be identified through the superscript “*c*”.

The mass balance equation in the reaction side is

(78) $$\frac{\partial \left(v_{z}c_{i}\right)}{\partial z}=\varepsilon \frac{1}{r}\frac{\partial }{\partial r}\left(rD_{ei}\frac{\partial c_{i}}{\partial r}\right)+\rho_{b}\left(1-\varepsilon \right)\sum_{j=1}^{N_{r}}\nu_{ij}r_{j}$$

where ε and $\rho_{b}$ are the porosity and density of the catalyst bed, respectively, $D_{ei}$ is the effective radial dispersion coefficient, and $N_{R}$ is the number of reactions. The boundary conditions proposed are

(79) $${\left.c_{i}\right|}_{z=0}=c_{i}^{in}$$

(80) $${\left.\frac{\partial c_{i}}{\partial r}\right|}_{r=0}=0$$

(81) $${\left.\left(D_{ei}\varepsilon \frac{\partial c_{i}}{\partial r}\right)\right|}_{r=R_{1}}={\left.\left(D_{ei}^{c}\varepsilon^{c}\frac{\partial c_{i}^{c}}{\partial r}\right)\right|}_{r=R_{1}}$$

The velocity of the gas mixture in the reaction side is evaluated from

(82) $$\frac{\partial v_{z}}{\partial z}=\frac{2R_{c}RT}{R_{1}^{2}P_{0}}\phi \left(\sqrt{p_{H_{2}}}-\sqrt{p_{H_{2}}^{c}}\right)+\frac{2RT}{R_{1}^{2}P_{0}}\rho_{b}\left(1-\varepsilon \right)\int_{r=0}^{r=R_{1}}\sum_{ij}\nu_{ij}r_{j}rdr$$

where $R_{c}$ is the outer radius of the ceramic support, R and is the gas constant.

The effective radial dispersion coefficient is evaluated from an expression of the form

(83) $$D_{ei}=AD_{mi}+BD_{mi}ReSc$$

where $D_{mi}$ is the molecular diffusion coefficient of the i−th component, evaluated as a function of gas composition

(84) $$D_{mi}=\frac{1-y_{i}}{\sum_{j=1,j\neq i}^{n}\left(y_{h}/D_{i,j}\right)}$$

Diffusion is the only mass transport mechanism in the ceramic support so the mass balance equation reads

(85) $$\frac{\varepsilon^{c}}{r}\frac{\partial }{\partial r}\left(rD_{ei}\frac{\partial c_{i}^{c}}{\partial r}\right)=0$$

and since only hydrogen permeates across the membrane, the boundary conditions in $R_{c}$ are

(86) $${\left.\frac{\partial c_{i}^{c}}{\partial r}\right|}_{r=R_{c}}=0\;\;\;i\neq H_{2}$$

(87) $$-{\left.\left(D_{eH_{2}}^{c}\varepsilon^{c}\frac{\partial c_{H_{2}}^{c}}{\partial r}\right)\right|}_{r=R_{c}}=\phi \left(\sqrt{p_{H_{2}}^{c}}-\sqrt{p_{H_{2}}^{p}}\right)$$

where $p_{H_{2}}^{p}$ is the hydrogen partial pressure in the permeate side.

In the permeate side the gas flow is co-current to the shell side and the hydrogen concentration and velocity profiles are described through a 1D model as

(88) $$\frac{\partial \left(v_{z}^{p}c_{H_{2}}^{p}\right)}{\partial z}=0$$

(89) $$\frac{\partial v_{z}^{p}}{\partial z}=\frac{2R_{c}RT}{R_{1}^{2}P_{0}}\phi \left(\sqrt{p_{H_{2}}^{c}}-\sqrt{p_{H_{2}}^{p}}\right)$$

The energy balance equations were written by considering convective transport in the axial direction and conduction in the radial direction. In the tube side the energy balance equation reads

(90) $$\rho_{g}C_{p}v_{z}\frac{\partial T}{\partial z}=\frac{1}{r}\frac{\partial }{\partial r}\left(k_{er}r\frac{\partial T}{\partial r}\right)+\rho_{b}\left(1-\varepsilon \right)\sum_{j=1}^{N_{R}}r_{j}\left(-\Delta H_{j}\right)$$

and the boundary conditions that apply are

(91) $${\left.T\right|}_{z=0}=T_{in}$$

(92) $${\left.T\right|}_{r=R_{1}}={\left.T^{c}\right|}_{r=R_{1}}$$

(93) $${\left.\left(k_{er}\frac{\partial T}{\partial r}\right)\right|}_{r=R_{1}}={\left.\left(k^{c}\frac{\partial T^{c}}{\partial r}\right)\right|}_{r=R_{1}}$$

where $k_{er}$ is the effective heat conductivity in the packed bed and $k^{c}$ is the heat conductivity of the ceramic shell.

The energy balance equation in the ceramic support is

(94) $$\frac{1}{r}\frac{\partial }{\partial r}\left(k^{c}r\frac{\partial T^{c}}{\partial r}\right)=0$$

with the additional boundary condition

(95) $${\left.\left(k^{c}\frac{\partial T^{c}}{\partial r}\right)\right|}_{r=R_{c}}=\alpha_{1}\left(T^{p}-T^{c}\right)$$

where $T^{p}$ is the temperature in the permeate side, which is taken to be constant.

After validating the model against experimental results reported in the literature, the authors use it to investigate the influence of the operating conditions on the performance of the system. The approach followed in the two works described above allows for two observations. The first one regards one of the advantages of the development of a complete model, accounting for transport of mass, momentum, and energy in two dimensions. The fact that the models were validated against experimental data obtained by different groups testifies to the idea that a complete model is also versatile, and can be applied even to describe situations different from those for which the model was originally intended. On the other hand, to determine the effect of the operating parameters on the behavior of the system using the model developed, it is necessary to run the entire simulation because the complexity of the governing equations—and of their mutual dependence—is such that it is difficult to make predictions without solving them in their entirety.

## 10. Models with Different Degrees of Complexity

Although in most instances models are developed with the specific aim of describing and interpreting experimental data, some groups have explicitly tackled the issue of developing models tailored to either investigate a particular phenomenon or provide a tool for the prediction of the behavior of membrane reactors over a wide range of parameters. For instance, the authors of this work have thoroughly investigated the interplay between mass transport within the packed bed, permeation across the membrane, and reaction over a wide range of operating conditions for methane reforming membrane reactors [54,59,68,136,137]. In view of this objective, the following simplifying assumptions have been made:

- Equilibrium composition of the inlet feed
- Zero hydrogen partial pressure in the permeate
- Uniform temperature
- Negligible membrane inhibition

**Figure 6:**
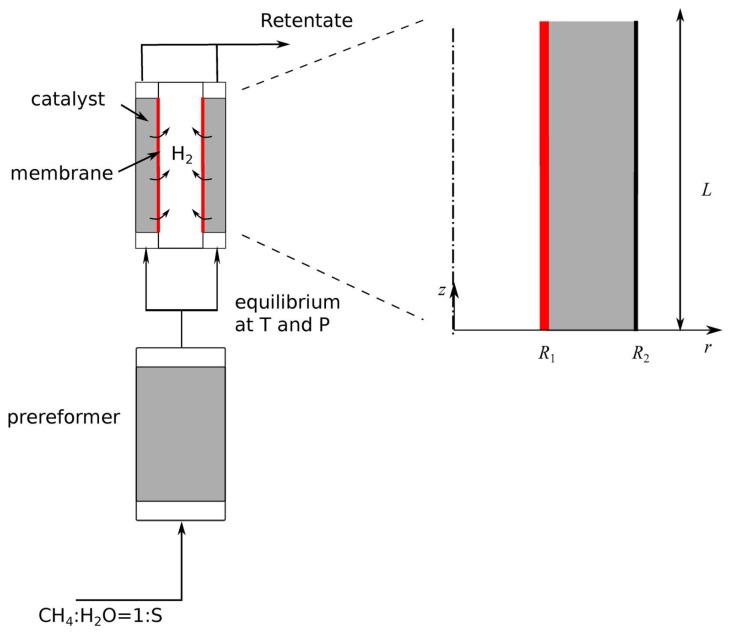
Schematic representation of the problem discussed in [54,59,68,136,137]. Reprinted from [59] with permission by Elsevier.

A schematic representation of the problem is shown in Figure 6.

The first two assumptions actually coincide with choices on the operating conditions. The first is equivalent to envisaging the presence of a traditional pre-reforming reactor before the membrane reactor and was motivated by the observation that the initial length of the membrane would be useless should the feed consist exclusively of methane and steam [53,55,138]. This is particularly important since the Pd-based membranes are often the most expensive component of membrane reactors. The second assumption corresponds to the use of either a high sweep flow rate or vacuum conditions in the permeate side. The third and fourth assumptions have been found to be physically justifiable. With regards to temperature, similar considerations to those made by Pieterse et al. [84] and reported in Section 7, were made and concerned the virtual absence of the temperature drop at the inlet of the reactor if the feed is the mixture obtained from a pre-reformer in which equilibrium conditions are reached. Furthermore, membrane reactors are designed to be heated by using molten salts for heat transport and storage [10,139] and previous studies have shown that an adequate design of the heating system allows a uniform temperature profile within the reactor [10,57]. On the other hand, the issue of membrane inhibition is important and worthy of being studied [5,20,140], but beyond the scope of the work carried out by the authors.

The model was developed for steady state conditions. Darcy’s law was used to describe the gas flow through the packed bed and the reaction rate was described through the expression proposed by Wei and Iglesia [70] and discussed in Section 7.1.1. It should be noted that $r_{i}$ is an effective reaction rate per unit volume of catalyst bed for which the efficiency factor is considered to be independent of the gas composition and is therefore implicitly included in the reaction rate constant. The total reforming reaction was considered, with the explanation that under the temperature conditions commonly adopted in membrane reactors the water-gas shift reaction can be considered to be completely shifted towards the products. The ensuing balance equations and corresponding boundary conditions are

(96) $$\nabla \cdot \left(\rho v\right)=0$$

(97) $$v=-\frac{\kappa }{\mu }\nabla P$$

(98) $$\nabla \cdot \left(-\frac{\kappa }{\mu }fP\;\omega_{i}\nabla P-fP\;D\cdot \nabla \omega_{i}\right)=r_{i}$$

(99) $${\left.v\right|}_{z=0}=v_{z}=U_{in}$$

(100) $${\left.\omega_{i}\right|}_{z=0}=\omega_{i}^{0,eq}$$

(101) $${\left.P\right|}_{z=L}=P_{L}$$

(102) $${\left.\frac{\partial \omega_{i}}{\partial z}\right|}_{z=L}=0$$

(103) $${\left.\frac{1}{RT}\left(-\frac{\kappa }{\mu }\nabla P\right)\right|}_{r=R_{1}}\cdot n_{1}=P_{m}\left(\sqrt{\frac{\omega_{h}}{W_{h}fP}}\right)$$

(104) $${\left.\frac{1}{RT}\left(-\frac{\kappa }{\mu }\omega_{h}\nabla P-D\cdot \nabla \omega_{h}\right)\right|}_{r=R_{1}}\cdot n_{1}=P_{m}\left(\sqrt{\frac{\omega_{h}}{W_{h}fP}}\right)$$

(105) $${\left.\frac{1}{RT}\left(-\frac{\kappa }{\mu }\omega_{i}\nabla P-D\cdot \nabla \omega_{i}\right)\right|}_{r=R_{1}}\cdot n_{1}=0\;,\;i\neq h$$

(106) $${\left.\nabla P\right|}_{r=R_{2}}\cdot n_{2}=0$$

(107) $${\left.\frac{1}{RT}\left(-\frac{\kappa }{\mu }\omega_{i}\nabla P-D\cdot \nabla \omega_{i}\right)\right|}_{r=R_{2}}\cdot n_{2}=0$$

Three dimensionless parameters groups were identified, namely γ, Pe, and Da. The first parameter

(108) $$\gamma =\frac{P_{m}RTP_{atm}^{-1/2}}{W_{h}}\frac{1}{U}$$

accounts for the ratio between the permeation velocity and the axial convection velocity and is proportional to the reciprocal of the membrane Peclet number, as defined by Patrascu and Sheintuch in [5].

The Peclet number

(109) $$\mathrm{Pe}=\frac{UR_{1}}{D}$$

represents the ratio between the characteristic times of diffusion and convection, and the Damkholer number

(110) $$\mathrm{Da}=\frac{RTkR_{1}}{U}$$

represents the ratio between the characteristic times of convection and reaction.

**Figure 7:**
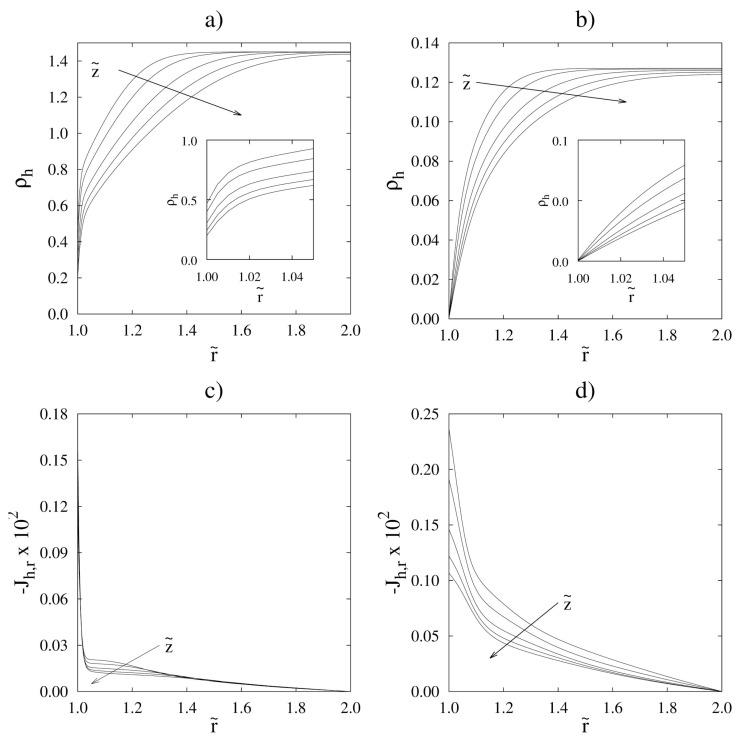
Radial profiles of the (dimensionless) hydrogen density (**a**,**b**) and flux (**c**,**d**) along the cross-section of a membrane reactor at different axial coordinates, *z*. The membrane wall is at $\tilde{r}=1$ and the impermeable wall is at $\tilde{r}=2$. The sign of the radial flux is negative because it is directed towards decreasing values of the radial coordinate. Panels (**a**) and (**d**) refer to the membrane-controlled regime (case 1). Panels (**b**) and (**d**) refer to the transport-controleld regimes (case 2). Reprinted from [55] with permission from Elsevier.

One of the main points of this series of works regards the importance of accounting for the radial velocity component and of the interplay between mass and momentum transport. As briefly mentioned earlier, the presence of a permeating flux necessarily causes the presence of a radial velocity component. By neglecting it, its influence on the radial dispersive transport is lost. Obviously, in some cases this loss of information may be insignificant, particularly when the membrane permeability is low or the mass dispersion is high; however, in other cases the error made on the concentration profiles by introducing this approximation translates into an error in the evaluation of integral quantities, such as the total hydrogen permeate flow rate and recovery, and consequently in the design of the reactors. In fact, a strict relationship between the behavior of local variables (i.e., concentration profiles) in proximity of the membrane and integral quantities has been shown in previous works [55,84] (see discussion of Figure 7 below).

After presenting and discussing their 2D models [54,59] along with the implications of the findings on design choices [55,136] the authors move on to consider four simplified scenarios and the corresponding models and tailored boundary conditions [54,68]

1. Infinitely fast reaction (i.e., Da>>1) and hydrogen permeation limited by transport across the membrane (case 1)
2. Infinitely fast reaction and hydrogen permeation limited by transport in the packed bed (case 2)
3. Infinitely slow reaction (i.e., Da<<1) and hydrogen permeation limited by transport across the membrane (case 3)
4. Infinitely slow reaction and hydrogen permeation limited by transport in the packed bed (case 4)

In cases 1 and 3 the behavior of the system is limited by transport across the membrane. Roughly speaking this situation is encountered when the membrane permeability (γ) is low and the dispersion (1/Pe) is high. Under these conditions the problem admits an analytical solution [54,68]. On the other hand, for cases 2 and 4 the problem requires the solution of a set of two ODEs. In all situations the aim is to evaluate integral quantities, specifically the hydrogen permeate flow rate, and the starting point is a simplified model in which axial dispersion is neglected with respect to axial convection, radial convection is neglected with respect to dispersion, the average molecular mass is considered constant along the reactor, and pressure drops are neglected. The boundary conditions and additional assumptions are then tailored to the specific scenario described.

In what follows we focus on a discussion of cases 1 and 2, which are more within the scope of the present work because the presence of a reaction is considered. A detailed discussion of the last two cases may be found in [54]. The variables that appear in the following paragraphs are in dimensionless form, but their meaning is easily understandable and follows the notation used in the rest of this work, unless otherwise stated.

Figure 7 shows that, in the presence of an inifinetely fast reaction, depending on whether the reactor is operating in the membrane-controlled (panels (a) and (c)) or transport-controlled (panels (b) and (d)) regime, the hydrogen concentration profiles change significantly. In the first case, the concentration on the membrane is different from zero and the total radial hydrogen flux is low in the entire reactor, with the exception of a small boundary layer close to the membrane wall. In the latter case, instead, the hydrogen concentration drops to zero as the membrane is approached and the high concentration gradients along the reactor’s cross-section result in significant radial fluxes of hydrogen.

For case 1 the mass balance equations for the non-permeating i−th component and for hydrogen reduce, respectively, to the following 1D equations

(111) $$-\frac{d\rho_{i}}{dz}+\nu_{i}Da\rho_{CH_{4}}\left(1-\eta \right)=0\;\;\;i\neq H_{2}$$

(112) $$-\frac{d\rho_{H_{2}}}{dz}+\nu_{H_{2}}Da\rho_{CH_{4}}\left(1-\eta \right)=2\gamma \frac{1}{\sigma^{2}-1}\sqrt{\rho_{H_{2}}}$$

Their solution yields the average concentration profiles along the reactor. The permeate flow rate may be evaluated from simple mass balance considerations as

(113) $$\Pi_{H_{2}}=\pi \left(\sigma^{2}-1\right)\left[\underset{\begin{array}{c} \mathrm{inlet}\;H_{2}\\ \mathrm{concentration} \end{array}}{\underset{︸}{\rho_{H_{2}}^{0}}}-\underset{\begin{array}{c} \mathrm{outlet}\;H_{2}\\ \mathrm{concentration} \end{array}}{\underset{︸}{\rho_{H_{2}}^{out}}}-\underset{H_{2}\;\mathrm{produced}\;\mathrm{by}\;\mathrm{reaction}}{\underset{︸}{\frac{\nu_{H_{2}}}{\nu_{CH_{4}}}\left(\rho_{CH_{4}}^{0}-\rho_{CH_{4}}^{out}\right)}}\right]$$

where σ is the ratio between the outer and inner radii.

If transport in the packed bed is considered to be the factor limiting the performance of the system (case 2), a 2D balance is required; however, it is sufficient to consider the mass balance equations of only hydrogen and a second component, such as methane, which read

(114) $$-\frac{\partial \rho_{H_{2}}}{\partial z}+\frac{1}{Pe_{eff,r}}\left(\frac{1}{r}\frac{\partial \rho_{H_{2}}}{\partial r}+\frac{\partial^{2}\rho_{H_{2}}}{\partial r^{2}}\right)+\nu_{H_{2}}Da\rho_{CH_{4}}\left(1-\eta \right)=0$$

(115) $$-\frac{\partial \rho_{CH_{4}}}{\partial z}+\frac{1}{Pe_{eff,r}}\left(\frac{1}{r}\frac{\partial \rho_{CH_{4}}}{\partial r}+\frac{\partial^{2}\rho_{CH_{4}}}{\partial r^{2}}\right)+\nu_{CH_{4}}Da\rho_{CH_{4}}\left(1-\eta \right)=0$$

where $Pe_{eff,r}$ is the effective Peclet number in the radial direction.

By introducing the auxiliary variable

(116) $$\Omega =-\nu_{CH_{4}}\rho_{CH_{4}}+\nu_{H_{2}}\rho_{CH_{4}}$$

the problem may be rewritten as

(117) $$-\frac{\partial \Omega }{\partial z}+\frac{1}{Pe_{eff,r}}\left(\frac{1}{r}\frac{\partial \Omega }{\partial r}+\frac{\partial^{2}\Omega }{\partial r^{2}}\right)=0$$

The inlet boundary condition may be simply derived from the combination of the inlet hydrogen and methane concentrations. Similarly, the condition of impermeability on the outer wall holds true even for the quantity described by the variable Ω. The issue of the boundary condition on the membrane is solved by considering that if the membrane resistance is negligible, the hydrogen concentration on the membrane goes to zero and the methane concentration must also go to zero, in light of the reaction equilibrium conditions. Therefore one has the following boundary conditions

(118) $${\left.\Omega \right|}_{z=0}=\Omega^{0}$$

(119) $${\left.\Omega \right|}_{r=R_{1}}=0$$

(120) $${\left.\frac{\partial \Omega }{\partial r}\right|}_{r=R_{2}}=0$$

In cylindrical coordinates, the solution of this problem, and the resulting permeate flow rate, may be expressed through the use of the Bessel functions [68,141].

(121) $$\Omega =\Omega^{0}\;\sum_{n=1}^{\infty }\frac{1}{N\left(\lambda_{n}\right)}exp\left(-\varepsilon \lambda_{n}^{2}z\right)\;R_{0}{\left(\lambda_{n},r\right)}_{r=1}^{\sigma }rR_{0}\left(\lambda_{n},r\right)dr$$

and

(122) $$\Pi_{h}=\frac{2\pi }{-\nu_{m}}\Omega^{0}\;g\left(Pe\right)$$

where g(Pe) is a function that accounts for the dispersive contribution to mass transport and depends only on the Peclet number and on the geometrical characteristics of the reactor, namely the aspect ratio $\left(L/R_{1}\right)$ and the characteristic dimension of the packing $\left(\delta \right)$. The value of g(Pe) decreases with increasing values of the Peclet number and tends to a constant value as Pe tends to zero. For high values of Pe, the function g(Pe) saturates towards a value that depends on δ; however, in typical operating conditions this saturation limit is hardly ever reached.

The Bessel functions appearing in Equations (121) and (122) are

(123) $$\frac{1}{N\left(\lambda_{n}\right)}=\frac{\pi^{2}}{2}\frac{\lambda_{n}^{2}J_{0}^{2}\left(\lambda_{n}\right)}{J_{0}^{2}\left(\lambda_{n}\right)-J_{1}^{2}\left(\lambda_{n}\sigma \right)}$$

(124) $$R_{0}\left(\lambda_{n},r\right)=J_{0}\left(\lambda_{n}r\right)Y_{0}^{\prime }\left(\lambda_{n}\sigma \right)-J_{0}^{\prime }\left(\lambda_{n}\sigma \right)Y_{0}\left(\lambda_{n}r\right)$$

$\lambda_{n}$ are the roots of

(125) $$J_{0}\left(\lambda_{n}\right)Y_{0}^{\prime }\left(\lambda_{n}\sigma \right)-Y_{0}\left(\lambda_{n}\right)J_{0}^{\prime }\left(\lambda_{n}\sigma \right)=0$$

The smallest zero of Equation (125) may be found in [142] for values of σ ranging from 1 to 20.

**Figure 8:**
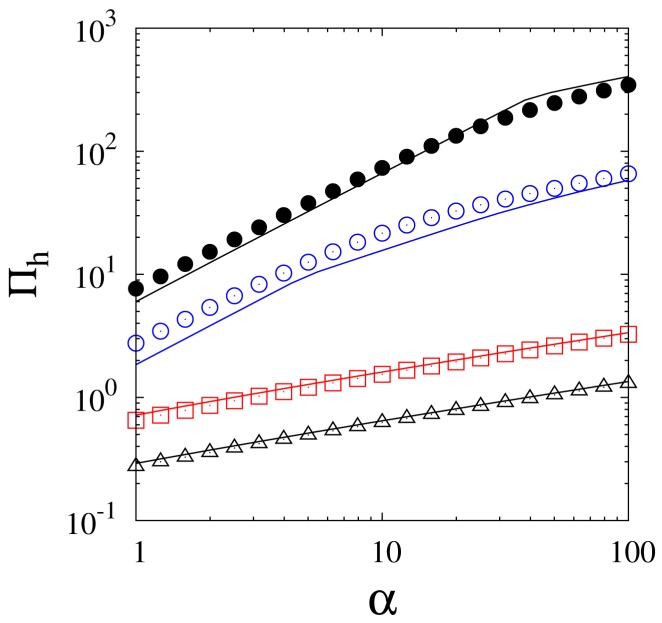
Comparison between model predictions (continuous lines) and results of the fully coupled transport model (symbols) at Da=10 and different values of γ and Pe. (black) solid circles: γ=3, Pe=10; (blue) empty circles: $\gamma =5\times 10^{-1}$, $Pe=1\times 10^{2}$; (red) empty squares: $\gamma =2\times 10^{-2}$, $Pe=2\times 10^{2}$; (black) empty triangles: $\gamma =8\times 10^{-3}$, $Pe=5\times 10^{2}$. Reprinted from [68] with permission from Elsevier.

The main novelty of this approach is that rather than using a unique simplified model, different solutions are proposed on the basis of the operating conditions, identified through a group of dimensionless parameters, thus providing a versatile tool for determining integral quantities with a good degree of accuracy and low computational cost under a wide range of operating conditions. A comparison between the results obtained from the simplified model and those derived from the fully coupled model is reported in Figure 8 [68]. Note that since the inlet composition and flow rate are known, the good agreement obtained for the permeate flow rate will also be observed for the hydrogen yield, defined as the ratio between the permeating hydrogen flux and the inlet methane flow rate.

**Figure 9:**
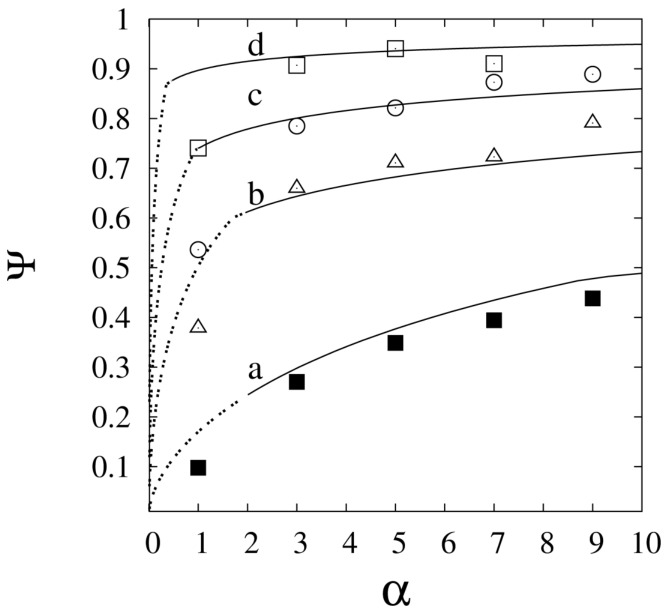
Recovery as predicted by the simplified model(lines) and measured experimentally (symbols) in [4] at $□:500\;{\mathrm{Ncm}}^{3}/\min$; $\circ :1000\;{\mathrm{Ncm}}^{3}/\min$; $▵:2000\;{\mathrm{Ncm}}^{3}/\min$; $▪:8000\;{\mathrm{Ncm}}^{3}/\min$. Reprinted from [68] with permission from Elsevier.

The simplified model was also validated against the experimental data reported in [4]. Figure 9 shows the recovery (Ψ), defined as the ratio between the hydrogen flow rate across the membrane and the total hydrogen produced by the reaction, as determined through the simplified model (lines) and as measured experimentally (points). Each curve refers to a different flow rate. At constant inlet flow rate the permeation is limited by transport across the membrane at low pressure and transport in the packed beds at higher pressures. The transition between the two regimes has been highlighted in the figure by changing the style of the line from dashed to solid. A more detailed explanation of the figure may be found in the original reference [68].

The use of the simplified model to determine optimal hydrogen yield in a range of operating conditions has been reported in [136], whereas the effects of reactor dimensions have been discussed in [55].

To summarize the findings of the works described above, we illustrate an example of their use. Let us suppose that we want to determine the optimal working pressure for an existing reactor, i.e., knowing the characteristics of the membrane and catalyst, the reactor dimensions, and the operating temperature. To estimate the hydrogen permeate flow rate as a function of pressure the following procedure could be followed, provided that the reaction is not the rate-limiting process

1. Determine the value of the dimensionless groups (Pe, γ, Da);
2. Evaluate the permeate flow rate as a function of pressure with Equation (113) (membrane-controlled regime);
3. Evaluate the permeate flow rate as a function of pressure with Equation (122) (transport-controlled regime);
4. Draw on the same graph the curves depicting the permeate flow rate as a function of pressure obtained in steps 2 and 3;
5. The “actual” permeate flow rate will be given, for any pressure value, by the lowest of the two curves

Another example, in which the need to consider a complex model has been explicitly dealt with, is represented by the work of Sheintuch [76], in which the discussion is motivated by the observation that the observed permeance of membranes employed in integrated reactors is often significantly lower than the values measured in experiments using pure hydrogen. This phenomenon is generally ascribed to two main factors: concentration polarization and membrane inhibition by competitive adsorption, as discussed in Section 3 of the present work. When concentration polarization is important, radial gradients cannot be neglected in the description of the system, and a 2D model is required. To identify the conditions in which 2D models are necessary, the author of [76] initially defines a parameter representing the ratio between the maximum hydrogen transport across the packed bed and the maximum hydrogen permeation across the membrane

(126) $$\Gamma =\frac{\varepsilon D_{er}\sqrt{P}}{v_{m,ref}RP_{ref}}$$

where $v_{m,ref}$ is a reference velocity describing hydrogen permeation across the membrane and *R* is the characteristic dimension of the reactor. In a previous work [69] it had been concluded that if 4Γ>>1 radial gradients may be neglected. If this condition is not met, radial gradients are significant and must be accounted for. Rather than employing a full 2D model, Sheintuch [76] proposes the use of an *effectiveness factor* that reduces the theoretical value of the flux across the membrane. The effectiveness factor is defined as

(127) $$\eta ={\left(1+\frac{1}{n\Gamma }\frac{1-<y_{H}>}{\sqrt{<y_{H}>}+\sqrt{P^{p}y_{h}^{p}/P}}\right)}^{-1}$$

where n=4 if the catalyst is placed in a cylindrical volume and n=3 if the catalyst is placed in the annulus, $<y_{H}>$ is the average hydrogen concentration in the reacting side and the superscript “*p*” refers to variables in the permeate side. The expression for η was derived from considerations carried out on a simplified model, in which the radial concentration profile of hydrogen is approximated through a quadratic equation and radial convection is neglected.

By comparing the model results with experimental data, the author concludes that concentration polarization is not sufficient to justify the observed permeance drop, but inhibition must also be taken into account. This motivates the subsequent atomistic model aimed at determining the influence of the different species present in the reforming mixture on membrane inhibition.

## 11. Concluding Remarks and Directions of Future Work

Ther present work has been directed towards a critical analysis of model development for the description of membrane reactors for hydrogen production, with particular reference to steam reforming systems. The constitutive equations describing hydrogen flux across the membrane, the rates of reaction, and heat exchange between the reaction volume and the permeate and wall have been reported and discussed, as they represent important components of the model. Nonetheless, the main focus has been on the presentation of the models that may be constructed and on the motivations for and implications of the introduction of simplifying assumptions.

The literature review highlighted the wealth of models developed to describe the performance of systems that were also tested experimentally. Several works have emphasized the importance of understanding the interplay between different mass transport mechanisms on the overall performance of the system, and the different mechanisms have also been studied independently. This is evidenced by the works that single out and tackle issues such as concentration polarization, membrane permeability and inhibition, and reaction rate expressions. In this framework, research is missing on the characterization of mass transport in reactors in which the catalyst is deposited on solid foams or other structured mechanical supports, with the exception of a very limited number of studies [143,144,145].

In addition, an equally in-depth study of the interplay between the efficiency of heat transport and the performance of membrane reactors seems to be lacking. An interest in the accurate description of heat tranfer, both between the reactor wall and the packed bed, and within the packed bed, i.e., effective heat conductivity, has been rekindled with the proposal of use of structured catalyst supports. Their significantly different structure compared to that of traditional packed beds implies that both the solid and the gaseous phases in the reactor are continuous and has introduced the necessity of carrying out new studies and developing appropriate correlations for the description of heat transport.

## Figures and Tables

**Table 1:** Momentum transport equations.

(1) $$\frac{\partial }{\partial t}\left(\varepsilon \rho v\right)+\nabla \cdot \left(\varepsilon \rho vv\right)=\underset{a}{\underset{︸}{-\varepsilon \nabla P}}-\underset{b}{\underset{︸}{\beta \varepsilon \rho v}}-\underset{c}{\underset{︸}{\nabla \cdot \left(\varepsilon \bar{\bar{\tau }}\right)}}+\underset{d}{\underset{︸}{\varepsilon \rho g}}$$
(2) $$\beta =150\frac{{\left(1-\varepsilon \right)}^{2}}{\varepsilon^{3}}\frac{\mu }{\rho d_{p}^{2}}+1.75\frac{1-\varepsilon }{\varepsilon^{3}}\frac{\varepsilon \left|v\right|}{d_{p}}$$
(3) $$\bar{\bar{\tau }}=-\left(\lambda -\frac{2}{3}\mu \right)\left(\nabla \cdot \bar{v}\right)\bar{I}-\mu \left[\left(\nabla v\right)+{\left(\nabla v\right)}^{T}\right]$$
or:
(4) $$v=-\frac{\kappa }{\mu }\nabla P\;\kappa =\frac{\phi_{s}^{2}d_{p}^{2}}{150}\frac{\varepsilon^{3}}{{\left(1-\varepsilon \right)}^{2}}$$
Boundary conditions
(5a) $${\left.v\right|}_{z=0}=\left(v_{z}^{0},0\right)$$ (5b) $$\left.P_{z=L}\right|=P_{L}$$ (5c) $${\left.\left(\rho v_{r}\right)\right|}_{r=R_{1}}\cdot n_{1}=J_{h}^{m}$$ (5d) $${\left.\nabla P\right|}_{r=R_{2}}\cdot n_{2}=0$$

$\left|v\right|=\sqrt{v_{r}^{2}+v_{z}^{2}}$, μ: gas viscosity, $d_{p}$: characteristic dimension of the catalyst particles, λ: dilatational viscosity, **v**: superficial velocity, *P*: pressure κ: bed permeability, $\phi_{s}$: catalyst particle sphericity, $n_{1}$ and $n_{2}$: local unit vectors normal to the surface and oriented outward the reaction volume. The marked a−d in Equation (1) are defined in the main text.

**Table 2:** Mass transport equations.

(6a) $$\frac{\partial \rho }{\partial t}+\nabla \cdot \left(\rho v\right)=0$$
(6b) $$\rho \frac{\partial \omega_{i}}{\partial t}+\rho v\cdot \nabla \omega_{i}=\nabla \cdot \left(\rho D_{i}\cdot \nabla \omega_{i}\right)+M_{i}\sum_{j}\alpha_{i,j}r_{j}\;D_{i}=\left(\begin{array}{cc} D_{ei,r} & 0\\ 0 & D_{ei,z} \end{array}\right)$$
Boundary conditions
(7) $${\left.\left(\rho v_{z}\omega_{i}-\rho D_{ei,z}\frac{d\omega_{i}}{dz}\right)\right|}_{z=0}=\rho^{0}v_{z}^{0}\omega_{i}^{0}\;\mathrm{or}\;{\left.\omega_{i}\right|}_{z=0}=\omega_{i}^{0}$$ (8) $${\left.\rho D_{ei,z}\frac{d\omega_{i}}{dz}\right|}_{z=L}=0$$ (9) $${\left.\left(\rho v_{r}\omega_{i}-\rho D_{ei,r}\frac{\partial \omega_{i}}{\partial r}\right)\right|}_{r=R_{1}}\cdot n_{1}=0\;\;\;\;\mathrm{for}\;i\neq H$$ (10) $${\left.\left(\rho v_{r}\omega_{i}-\rho D_{ei,r}\frac{\partial \omega_{i}}{\partial r}\right)\right|}_{r=R_{1}}\cdot n_{1}=J_{h}^{m}$$ (11) $${\left.\left(\rho v_{r}\omega_{i}-\rho D_{ei,r}\frac{\partial \omega_{i}}{\partial r}\right)\right|}_{r=R_{2}}\cdot n_{2}=0$$

$n_{1}$ and $n_{2}$: local unit vectors normal to the surface and oriented outward the reaction volume.

**Table 3:** Mass transport equations in molar units.

(12a) $$\frac{\partial c_{tot}}{\partial t}+\nabla \cdot \left(c_{tot}v^{*}\right)=\sum_{i,j}\alpha_{i,j}r_{j}$$
(12b) $$c_{tot}\frac{\partial x_{i}}{\partial t}+c_{tot}v^{*}\cdot \nabla x_{i}=c_{tot}\nabla \cdot \left(D_{i}\cdot \nabla x_{i}\right)+\sum_{j}\alpha_{i,j}r_{j}\;D_{i}=\left(\begin{array}{cc} D_{ei,r} & 0\\ 0 & D_{ei,z} \end{array}\right)$$
Boundary conditions
(13) $${\left.\left(c_{tot}v_{z}^{*}x_{i}-c_{tot}D_{ei,z}\frac{dx_{i}}{dz}\right)\right|}_{z=0}=0\;\mathrm{or}\;{\left.x_{i}\right|}_{z=0}=x_{i}^{0}$$ (14) $${\left.c_{tot}D_{ei,z}\frac{dx_{i}}{dz}\right|}_{z=L}=0$$ (15) $${\left.\left(c_{tot}v_{r}^{*}x_{i}-c_{tot}D_{ei,r}\frac{\partial x_{i}}{\partial r}\right)\right|}_{r=R_{1}}\cdot n_{1}=0\;\;\;\;\mathrm{for}\;i\neq H$$ (16) $${\left.\left(c_{tot}v_{r}^{*}x_{h}-c_{tot}D_{eh,r}\frac{\partial x_{h}}{\partial r}\right)\right|}_{r=R_{1}}\cdot n_{1}=J_{h}^{*m}$$ (17) $${\left.\left(c_{tot}v_{r}^{*}x_{i}-c_{tot}D_{ei,r}\frac{\partial x_{i}}{\partial r}\right)\right|}_{r=R_{2}}\cdot n_{2}=0$$

$v^{*}$: molar-averaged velocity, $c_{tot}$: total molar concentration, $x_{i}$: molar fraction, $J_{h}^{*m}$: molar flux of hydrogen across the membrane, $n_{1}$ and $n_{2}$: local unit vectors normal to the surface and oriented outward the reaction volume.

**Table 4:** Energy transport equations.

(18) $${\left(\rho c_{p}\right)}_{bed}\frac{\partial T}{\partial t}+{\left(\rho c_{p}\right)}_{gas}v\cdot \nabla T=k_{e}\nabla^{2}T-\sum_{j}r_{j}\left(-\Delta H_{j}\right)$$
Boundary conditions
(19) $${\left.\left(v_{z}\rho c_{p}T-k_{e,z}\frac{dT}{dz}\right)\right|}_{z=0}=v_{z}^{0}\rho^{0}c_{p}T_{in}\;\mathrm{or}\;{\left.T\right|}_{z=0}=T_{in}$$ (20) $${\left.k_{e,z}\frac{dT}{dz}\right|}_{z=L}=0$$ (21) $${\left.\left(v_{r}\rho c_{p}T-k_{e,r}\frac{\partial T}{\partial r}\right)\right|}_{r=R_{1}}\cdot n_{1}={\dot{Q}}_{permeate}$$ (22) $${\left.\left(v_{r}\rho c_{p}T-k_{e,r}\frac{\partial T}{\partial r}\right)\right|}_{r=R_{2}}\cdot n_{2}={\dot{Q}}_{wall}$$

$c_{p}$: heat capacity of the gas, $k_{e}$: effective heat conductivity of the packed bed, $r_{j}$: rate of the j-th reaction, $\Delta H_{j}$: associated heat of reaction, $n_{1}$ and $n_{2}$: local unit vectors normal to the surface and oriented outward the reaction volume.

**Table 5:** 1D cross-section averaged equations of change.

(23a) $$\langle \frac{\partial \rho }{\partial t}\rangle =-\frac{\partial \langle \rho v_{z}\rangle }{\partial z}-{\left.\rho v_{r}\right|}_{m}a_{m}^{v}$$ (23b) $$\langle \rho \frac{\partial \omega_{i}}{\partial t}\rangle =-\frac{\partial \langle \rho v_{z}\omega_{i}\rangle }{\partial z}-\frac{\partial }{\partial z}\langle \rho D_{i,z}\frac{\partial \omega_{i}}{\partial z}\rangle +M_{i}\sum_{j}\langle \alpha_{ij}r_{j}\rangle \;\mathrm{for}\;i\neq H$$ (23c) $$\langle \rho \frac{\partial \omega_{h}}{\partial t}\rangle =-\frac{\partial \langle \rho v_{z}\omega_{h}\rangle }{\partial z}-\frac{\partial }{\partial z}\langle \rho D_{i,z}\frac{\partial \omega_{h}}{\partial z}\rangle +M_{h}\sum_{j}\alpha_{hj}\langle r_{j}\rangle -{\left.N_{h}\right|}_{m}a_{m}^{v}$$ (23d) $$\langle {\left(\rho c_{p}\right)}_{bed}\frac{\partial T}{\partial t}\rangle =-\frac{\partial \langle \rho v_{z}c_{p}T\rangle }{\partial z}-\frac{\partial }{\partial z}\langle k_{ez}\frac{\partial T}{\partial z}\rangle +\sum_{j}\langle -\Delta H_{j}r_{j}\rangle +{\dot{Q}}_{perm}a_{m}^{v}+{\dot{Q}}_{wall}a_{w}^{v}$$

**Table 6:** Methane steam reforming reactions.

(28a) $${\mathrm{CH}}_{4}+H_{2}O⇌\mathrm{CO}+3H_{2}$$ (28b) $$\mathrm{CO}+H_{2}O⇌{\mathrm{CO}}_{2}+H_{2}$$ (28c) $${\mathrm{CH}}_{4}+{\mathrm{CO}}_{2}⇌2\mathrm{CO}+2H_{2}$$ (28d) $${\mathrm{CH}}_{4}+2H_{2}O⇌{\mathrm{CO}}_{2}+4H_{2}$$ (28e) $${\mathrm{CH}}_{4}⇌C+H_{2}$$ (28f) $$2\mathrm{CO}⇌{\mathrm{CO}}_{2}+C$$ (28g) $$\mathrm{CO}+H_{2}⇌C+H_{2}O$$

**Table 7:** Ethanol steam reforming reactions.

(32a) $$C_{2}H_{5}\mathrm{OH}+H_{2}O⇌2\mathrm{CO}+4H_{2}$$ (32b) $$C_{2}H_{5}\mathrm{OH}+3H_{2}O⇌2{\mathrm{CO}}_{2}+6H_{2}$$ (32c) $$C_{2}H_{5}\mathrm{OH}⇌C_{2}H_{4}O+H_{2}$$ (32d) $$C_{2}H_{4}O⇌{\mathrm{CH}}_{4}+\mathrm{CO}$$ (32e) $$C_{2}H_{4}O+H_{2}O⇌2\mathrm{CO}+3H_{2}$$ (32f) $$C_{2}H_{5}\mathrm{OH}⇌\frac{1}{2}{\mathrm{CO}}_{2}+\frac{3}{2}{\mathrm{CH}}_{4}$$ (32g) $${\mathrm{CO}}_{2}+4H_{2}⇌{\mathrm{CH}}_{4}+2H_{2}O$$ (32h) $$2C_{2}H_{5}\mathrm{OH}⇌C_{3}H_{6}O+\mathrm{CO}+3H_{2}$$ (32i) $$C_{2}H_{5}\mathrm{OH}+2H_{2}⇌2{\mathrm{CH}}_{4}+2H_{2}O$$

## Abbreviations

The following abbreviations are used in this manuscript:

$a_{z}$	geometric ratio $L/R_{1}$
$c_{p}$	specific heat [J/molK] or [J/kgK]
*d*	characteristic dimension of packed bed particles $\left[m\right]$
$D_{rr}$	radial dispersion coefficient $\left[m^{2}/s\right]$
$D_{zz}$	axial dispersion coefficient $\left[m^{2}/s\right]$
D	diffusion coefficient $\left[m^{2}/s\right]$
D	effective dispersion tensor $\left[m^{2}/s\right]$
*f*	average molar weight [kg/mol]
$J_{h}^{m}$	hydrogen mass flux through the membrane $\left[kg_{H_{2}}/\left(m^{2}\cdot s\right)\right]$
$J_{h}^{m*}$	hydrogen molar flux through the membrane $\left[mol_{H_{2}}/\left(m^{2}\cdot s\right)\right]$
*k*	reaction rate constant $\left[mol/\left(m^{3}\cdot s\cdot Pa\right)\right]$
$k_{e}$	effective heat conductivity [W/mK]
$K_{eq}$	equilibrium constant $\left[Pa^{2}\right]$
*L*	reactor length [m]
n	unit vector normal to the membrane surface $\left[-\right]$
*P*	pressure [Pa]
$P_{i}$	partial pressure of the *i-th* component [Pa]
$P_{L}$	outlet pressure $\left[Pa\right]$
$P_{U}$	reference pressure $\left(\mu UR_{1}/\kappa \right)$ $\left[Pa\right]$
$P_{m}$	membrane permeability $\left[kg_{H_{2}}/\left(m^{2}\cdot s\cdot Pa^{0.5}\right)\right]$
${\dot{Q}}_{wall}$	heat flux between catalyst and reactor wall $\left[W/m^{2}\right]$
${\dot{Q}}_{permeate}$	heat flux between catalyst and permeate $\left[W/m^{2}\right]$
*r*	radial coordinate [m]
$r_{i}$	volume-specific mass rate of production of the *i-th* component $\left[kg/\left(m^{3}\cdot s\cdot Pa\right)\right]$
R	gas constant $\left[J/\left(mol\cdot K\right)\right]$
$R_{1}$	inner reactor radius [m]
$R_{2}$	outer reactor radius [m]
$R_{m}$	volume-specific molar rate of methane consumption $\left[mol/\left(m^{3}\cdot s\cdot Pa\right)\right]$
*T*	temperature [K]
*U*	inlet gas velocity $\left[m/s\right]$
$U_{m}$	heat transfer coefficient between permeate and retentate $\left[W/m^{2}K\right]$
$U_{w}$	heat transfer coefficient between wall and retentate $\left[W/m^{2}K\right]$
v	mass average velocity $\left[m/s\right]$
$v^{*}$	molar average velocity $\left[m/s\right]$
$W_{i}$	molar weight of the *i-th* component [kg/mol]
*z*	axial coordinate [m]
**Greek symbols**	
α	dimensionless outlet pressure $\left(P_{L}/P_{U}\right)$
β	ratio between characteristic and inlet velocities $\left(\kappa P_{atm}/\left(\mu R_{1}U\right)\right)\;\left[-\right]$
γ	dimensionless permeability parameter $\left(P_{m}RTP_{atm}^{-1/2}/W_{h}U\right)$
Γ	$2\gamma /\left(a_{r}^{2}-1\right)$
δ	membrane thickness [m]
η	proximity to reaction equilibrium $\left[-\right]$
κ	packed bed permeability $\left[m^{2}\right]$
μ	gas viscosity $\left[Pa\cdot s\right]$
$\Pi_{H_{2}}$	dimensionless permeate flow rate
ρ	gas density $\left[kg/m^{3}\right]$
$\rho_{i}$	density of the *i-th* component $\left[kg/m^{3}\right]$
σ	geometric ratio, $R_{2}/R_{1}$
τ	packed bed tortuosity factor $\left[-\right]$
$\nu_{i}$	stoichiometric coefficient of the *i-th* component $\left[-\right]$
Φ	intrinsic membrane permeability
$\omega_{i}$	mass fraction of the *i-th* component $\left[-\right]$
Ω	linear combination of hydrogen and methane densities $\left(-\nu_{m}{\tilde{\rho }}_{h}+\nu_{h}{\tilde{\rho }}_{m}\right)$ $\left[-\right]$
Ψ	hydrogen recovery (permeated hydrogen/produced hydrogen) $\left[-\right]$
$\Psi_{s}$	separator-base yield (permeated hydrogen/inlet hydrogen) $\left[-\right]$
Θ	inhibition factor
**Dimensionless parameters**	
Da	Damkholer number $\left(RTkR_{1}/U\right)$
${\mathrm{Da}}^{*}$	modified Damkholer number $\left(\mathrm{Da}/\Gamma \right)$
${\tilde{D}}_{rr}$	dimensionless radial dispersion $\left(D_{rr}/D\right)$
$\tilde{D}$	dimensionless dispersion tensor $\left(D/D\right)$
$l_{p}$	characteristic length of permeation $2\sqrt{\rho_{h}^{0}}/\Gamma$
Pe	Peclet number $\left(UR_{1}/D\right)$
$Pe_{m}$	molecular Peclet number $\left(Ud/D\right)$
$Pe_{r}^{eff}$	effective radial Peclet number $\left(Ud/D_{rr}\right)$
$Pe_{z}^{eff}$	effective axial Peclet number $\left(Ud/D_{zz}\right)$
Re	Reynolds number $\rho UR_{1}/\mu$
Sc	Schmidt number $\mu /\left(\rho D\right)$
**Subscripts**	
*c*	carbon dioxide
*h*	hydrogen
*m*	methane
*w*	water
*i*	*i-th* component
